# Dichloroacetate improves mitochondrial function, physiology, and morphology in FBXL4 disease models

**DOI:** 10.1172/jci.insight.156346

**Published:** 2022-08-22

**Authors:** Manuela Lavorato, Eiko Nakamaru-Ogiso, Neal D. Mathew, Elizabeth Herman, Nina Shah, Suraiya Haroon, Rui Xiao, Christoph Seiler, Marni J. Falk

**Affiliations:** 1 Mitochondrial Medicine Frontier Program, Division of Human Genetics, Department of Pediatrics, Children’s Hospital of Philadelphia, Philadelphia, Pennsylvania, USA.; 2 Department of Pediatrics, Perelman School of Medicine, University of Pennsylvania, Philadelphia, Pennsylvania, USA.; 3 Department of Biostatistics, Epidemiology and Informatics, Perelman School of Medicine, University of Pennsylvania, Philadelphia, Pennsylvania, USA.; 4 Aquatics Core Facility, The Children’s Hospital of Philadelphia, Philadelphia, Pennsylvania, USA.

**Keywords:** Genetics, Metabolism, Drug therapy, Genetic diseases, Mitochondria

## Abstract

Pathogenic variants in the human F-box and leucine-rich repeat protein 4 (*FBXL4*) gene result in an autosomal recessive, multisystemic, mitochondrial disorder involving variable mitochondrial depletion and respiratory chain complex deficiencies with lactic acidemia. As no FDA-approved effective therapies for this disease exist, we sought to characterize translational *C*. *elegans* and zebrafish animal models, as well as human fibroblasts, to study *FBXL4^–/–^* disease mechanisms and identify preclinical therapeutic leads. Developmental delay, impaired fecundity and neurologic and/or muscular activity, mitochondrial dysfunction, and altered lactate metabolism were identified in *fbxl-1(ok3741) C*. *elegans*. Detailed studies of a PDHc activator, dichloroacetate (DCA), in *fbxl-1(ok3741)* *C*. *elegans* demonstrated its beneficial effects on fecundity, neuromotor activity, and mitochondrial function. Validation studies were performed in *fbxl4^sa12470^* zebrafish larvae and in *FBXL4^–/–^* human fibroblasts; they showed DCA efficacy in preventing brain death, impairment of neurologic and/or muscular function, mitochondrial biochemical dysfunction, and stress-induced morphologic and ultrastructural mitochondrial defects. These data demonstrate that *fbxl-1*(*ok3741*) *C*. *elegans* and *fbxl4^sa12470^* zebrafish provide robust translational models to study mechanisms and identify preclinical therapeutic candidates for *FBXL4^–/–^* disease. Furthermore, DCA is a lead therapeutic candidate with therapeutic benefit on diverse aspects of survival, neurologic and/or muscular function, and mitochondrial physiology that warrants rigorous clinical trial study in humans with *FBXL4^–/–^* disease.

## Introduction

F-box and leucine-rich repeat protein 4 (*FBXL4*) mitochondrial disease is an autosomal recessive disorder that manifests with a highly variable breadth and severity of multisystem features, including encephalomyopathy, global developmental delay, epilepsy, dysmorphic features, skeletal abnormalities, hypertrophic cardiomyopathy, arrhythmias, liver dysfunction, visual impairment, hearing loss, bone marrow deficiencies, renal tubular acidosis requiring bicarbonate supplementation, and pronounced lactic acidemia (1–5). Forty-eight *FBXL4* pathogenic variants have been reported in 94 patients, making it one of the more common nuclear gene causes of primary mitochondrial disease (1–3, 5–13). While, *FBXL4* disease has relatively high prevalence, morbidity, and mortality (4), no FDA-approved, effective therapies, or cures are currently known for *FBXL4* disease (2).

While F-box proteins consist of multiple classes that generally localize in the nucleus to function in protein-protein interactions that contribute to ubiquitin-mediated proteolysis (14), the precise subcellular localization and physiologic functions of FBXL4 remain poorly understood. FBXL4 has been found to localize, at least in part, in the mitochondrial intermembrane space (1, 2). Furthermore, FBXL4 deficiency leads to variable levels of mitochondrial depletion and multiple respiratory chain (RC) complex deficiencies (1, 2). Mitochondrial DNA (mtDNA) content has also been reduced to variable extents in *FBLX4^–/–^* patient muscle tissue and fibroblast cells, with variable impairment of fibroblast cell mitochondrial morphology, membrane potential, and oxygen consumption capacity (2). However, the precise mechanisms by which FBXL4 deficiency disrupts mitochondrial structure and functions are not known. Preclinical modeling in translational animals and cells is important to gaining an improved understanding of *FBXL4^–/–^* disease mechanisms (15), identifying therapeutic targets, and prioritizing candidate drug treatments.

Here, we utilized simple invertebrate (*Caenorhabditis elegans*, worm) and vertebrate (*Danio* *rerio*, zebrafish) animal models generated in large-scale mutagenesis screens to pursue preclinical disease modeling of *FBXL4^–/–^* disease.

### C.

*elegans* and zebrafish are now considered very informative models that can be used to advance understanding of genetic diseases. Indeed, both *C*. *elegans* and zebrafish are widely used to understand a wide array of human disease mechanisms, due to their well-characterized genomes and high homology of most genes with their human counterparts (16–22). Both animal models may be used to mimic RC dysfunction, with relevant features to those seen in patients with primary mitochondrial, disease and offer unique experimental advantages for drug screening (23–26). Despite the potential relevance of studying *FBXL4* mitochondrial disease in mammals, the homozygous *Fbxl4*-knockout mouse model showed perinatal lethality, with low survival rate (15). In contrast, the homozygous *Fbxl4^–/–^* zebrafish and *C*. *elegans* models used in our study had high survival rates, allowing for multiple long-term physiological studies. These two animal models (*D*. *rerio* and *C*. *elegans*) were used to evaluate the effect of *FBXL4^–/–^* mutations on animal behavior and physiology as well as for cross-evolutionary species validation studies of dichloroacetate (DCA) treatment efficacy.

In particular, we identified, in the *Caenorhabditis* Genetics Center public repository (https://cgc.umn.edu/), a previously uncharacterized *C*. *elegans* mutant strain, VC3038, which contains a homozygous *fbxl-1(ok374)* allele involving a 707–base pair deletion in the *C02F5.7* (*fbxl-1*) gene (27). We also identified a zebrafish strain, *fbxl4^sa12470^* (homozygous for a missense mutation in the sa12470 allele), from the public Sanger repository (28). We characterized both the worm and zebrafish *FBXL4^–/–^* animal models for swimming activity, gross morphological phenotypes, and mitochondrial physiology; stressor sensitivity was only tested in the zebrafish model. In addition, mitochondrial function was evaluated in fibroblast cell lines from 2 humans with *FBXL4^–/–^* disease. Furthermore, screening of empiric drug therapies was performed in the *C*. *elegans* model of *FBXL4^–/–^* disease, resulting in prioritization of DCA, an activator of the mitochondrial pyruvate dehydrogenase complex (PDHc), as the lead therapeutic candidate. Detailed investigations of DCA were performed to evaluate its effects on behavior, neurologic and/or muscular activity, mitochondrial function, and lactate metabolism in the *C*. *elegans* model of *FBXL4* mitochondrial disease, with subsequent validation studies performed in zebrafish exposed to mitochondrial translation inhibition with chloramphenicol (CAP) and human fibroblasts from individuals with *FBXL4^–/–^* disease.

## Results

### FBXL4 protein and its key domains are conserved across evolutionarily distinct species.

**Figure 1:**
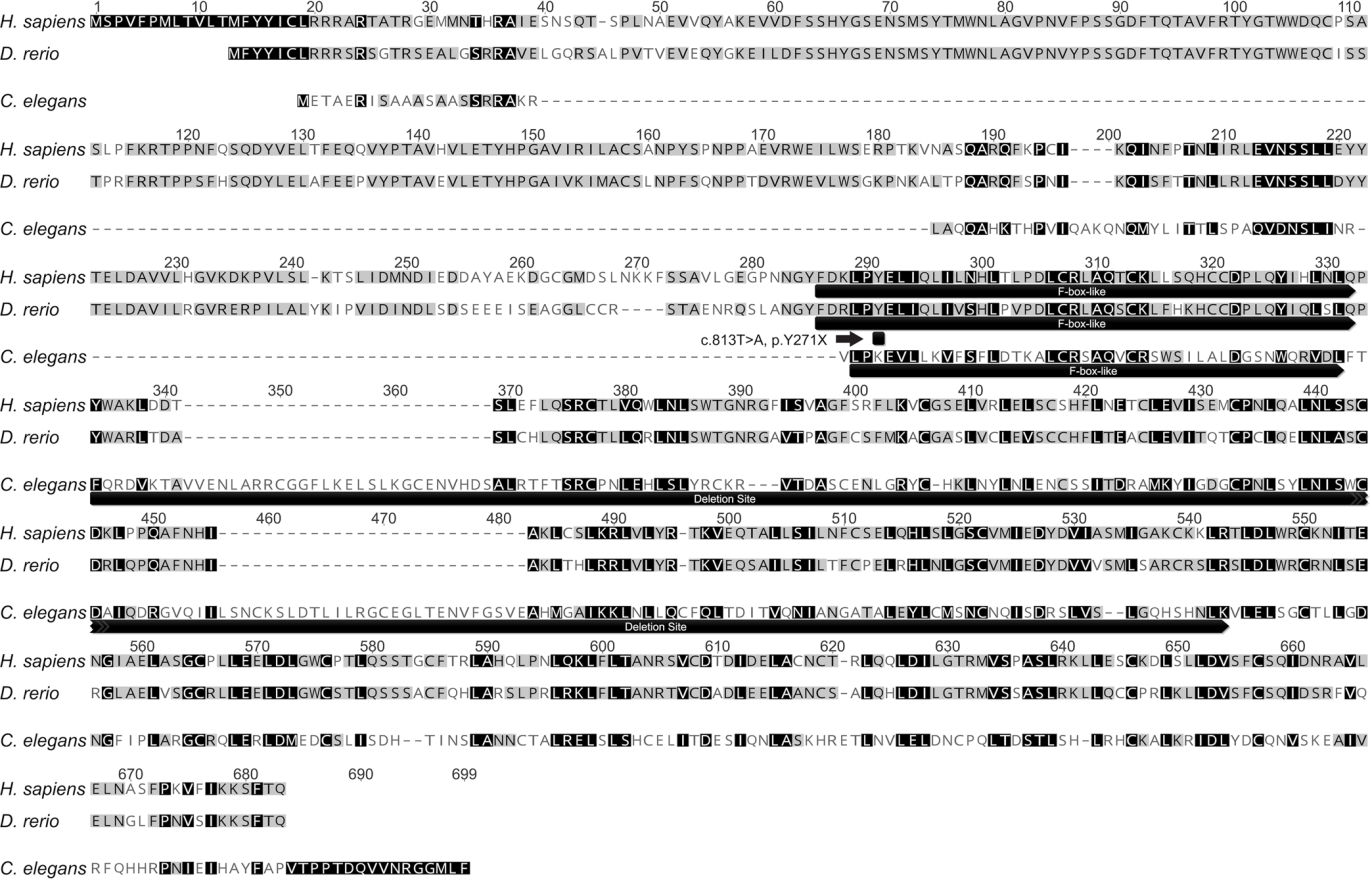
Protein alignment and evolutionary conservation of FBXL proteins in *Homo sapiens, D. rerio,* and *C. elegans*. Protein alignment of *H*. *sapiens*, *D*. *rerio,* and *C*. *elegans* (see Supplemental Figure 1 for alignment with other species). The locations of mutations in the zebrafish and *C*. *elegans* mutant strains studied are shown: c.813T>A, p.Y271X in zebrafish and g.410_1116del, p.Phe105_Lys308del in *C*. *elegans*. Protein alignment of conserved F-box–like domain among *H*. *sapiens*, *D*. *rerio*, and *C*. *elegans* is shown. Percentage homology is shown in gray, and white and black indicate the lowest and highest levels of similarity, respectively.

Sequence alignment of human FBXL4 protein showed a similarity of 80% with *D*. *rerio* (zebrafish) and 31% with *C*. *elegans* (worms) (Figure 1) and Supplemental Figure 1, which shows protein alignment with *Drosophila melanogaster*, *Mus musculus*, *D*. *rerio*, *C*. *elegans*). The mutation sites in both *FBXL4* disease models studied here are shown in Figure 1. The *fbxl4^sa12470^* zebrafish mutant line harbors a homozygous *T>A* missense allele in the F-box–like domain of zebrafish *fbxl4*. The *C*. *elegans* VC3038 strain harbors the *fbxl-1*(*ok3741)* allele that is a homozygous 707–base pair deletion immediately downstream of the F-box–like domain and extending across multiple leucine-like repeats. Interestingly, the human F-box–like domain, known to mediate protein-protein interactions, showed conservation across all species, with a similarity of 91.5% to *D*. *rerio* and 44.5% with *C*. *elegans* (Figure 1).

### fbxl-1(ok3741) C. elegans displayed developmental delay, abnormal growth, and reduced fecundity.

**Figure 2:**
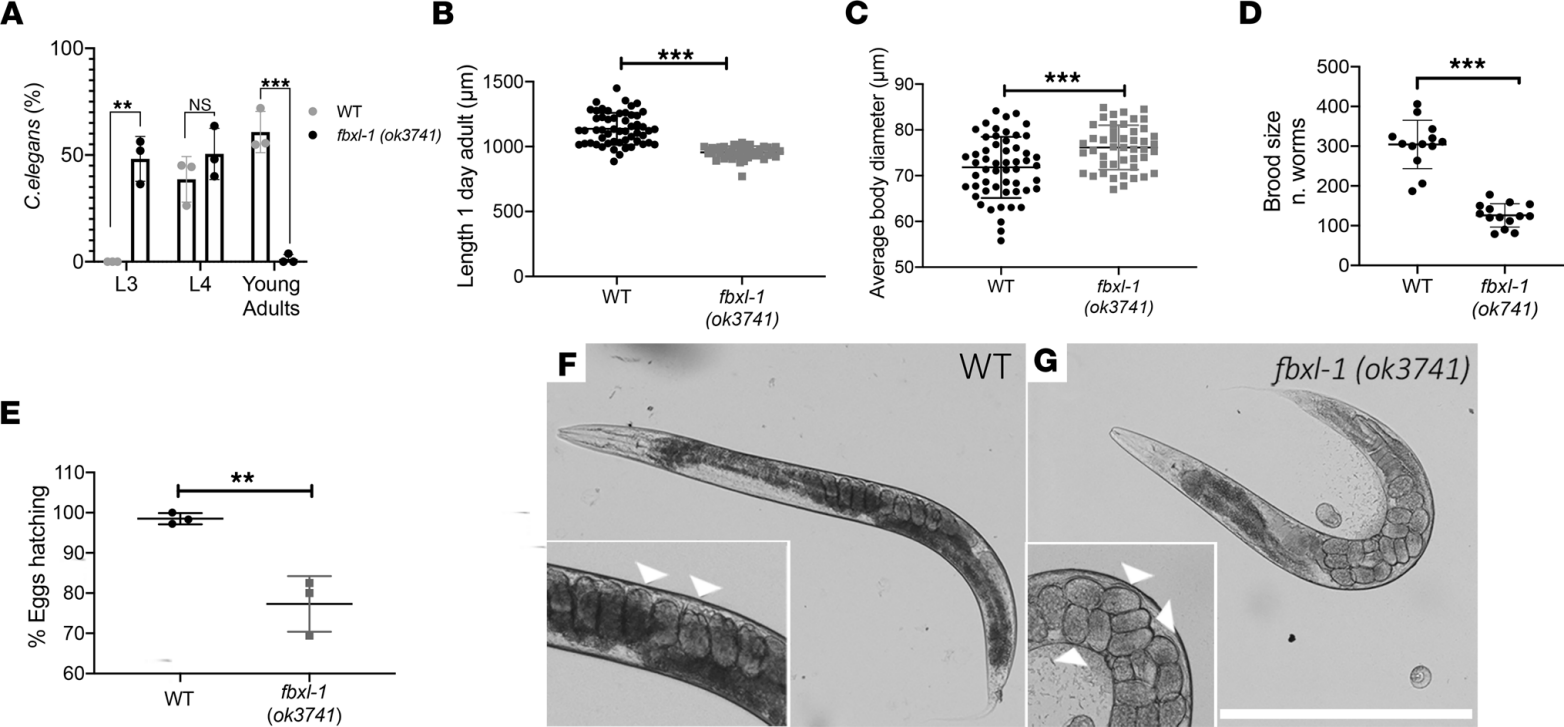
*fbxl-1(ok3741)* worms had abnormal development, growth, and fecundity. (**A**) At 64 hours after eggs were laid, 48% of *fbxl-1*(*ok3741)* worms had reached L3 larval development stage and 52% had reached the L4 larval stage. By contrast, 39% of N2 WT worms were at L4 larval development stage and 61% had reached the young adult stage, with no worms seen at the earlier larval stages. *n =* 168 N2 WT worms; *n =* 152 *fbxl-1(ok3741)* worms; 3 biological replicates. (**B** and **C**) *fbxl-1*(*ok3741)* worm average body length was significantly decreased, and averaged body width was significantly increased, compared with WT worms. *n* = 54 WT; *n* = 46 *fbxl-1*(*ok3741)*. (**D**) *fbxl-1*(*ok3741)* brood size was 58% smaller than that of N2 WT worms. *n* = 13 N2 WT adult worms; *n* = 14 *fbxl-1*(*ok3741)* adult worms; 3 biological replicates. (**E**) *fbxl-1*(*ok3741)* egg-hatching rate was 21% lower than that in N2 WT worms. *n =* 136 each strain; 3 biological replicates. (**F** and **G**) Images of WT and *fbxl-1*(*ok3741)* gravid worms at day 2 of the adult stage. *fbxl-1*(*ok3741)* gravid worms have disarranged eggs with differing polarity (**G**, arrowheads) as compared with WT gravid worms, in which eggs are organized in 1 or 2 layers with similar polarity (**F**, arrowheads). Data are shown as the mean ± SD. Statistical analyses were performed by Student’s *t* test. *P < 0.05, **P < 0.01, ***P < 0.001. Scale bar: 400 μm.

Larval development in *fbxl-1*(*ok3741)* worms was delayed by approximately 8 hours compared with N2 WT worms. Specifically, 64 hours after eggs were laid, when most of N2 WT worms had reached L4 and the young adult stage, *fbxl-1*(*ok3741*) worms had reached only the L3 and L4 larval stages (Figure 2A). Indeed at 64 hours after being laid, the L3 stage was observed for 0% of N2 WT worms versus 48.2% ± 10.5% (mean ± SD) of *fbxl-1(ok3741)* worms (*P <* 0.01), L4 stage was observed for 38.6% ± 10.7% of N2 WT worms versus 50.6% ± 12.0% of *fbxl-1(ok3741)* worms (not significantly different), and the young adult stage was observed for 60.8% ± 9.7% of N2 WT worms versus only 1.3% ± 2.2% of *fbxl-1(ok3741)* worms. Thus, *fbxl-1*(*ok3741*) worms displayed significant developmental delay in reaching adult stage.

*fbxl-1(ok3741)* worms also showed significant differences in body growth as compared with N2 WT control worms. Specifically, *fbxl-1(ok3741)* worm length at 1 day into young adult stage (955 ± 47 μm) was significantly reduced by 16% compared with that of N2 WT worms (1,137 ± 122 μm) (Figure 2B). Interestingly, the mean width of *fbxl-1(ok3741)* worms (76.2 ± 4.8 μm) was 6.1% larger than that of N2 WT worms (71.8 ± 6.7 μm) (Figure 2C).

Animal fecundity is an integrated reflection of both egg-laying and -hatching behaviors, as quantified by evaluation of the total number of progeny laid per worm (brood size analysis), which requires intact neuromuscular function of the vulva, as well as the hatch rate of eggs once laid (egg hatch rate). By counting the number of progeny obtained over the typical 6-day reproductive lifetime of young adult worms, the *fbxl-1*(*ok3741*) brood size (117 ± 30 larvae) was observed to be significantly reduced by 58% relative to the N2 WT brood size (283 ± 54 larvae) (Figure 2D). To better understand the mutant worm strain’s reduced reproductive capacity, egg hatch rates were also estimated. The *fbxl-1*(*ok3741*) worm egg hatch rate (77.3% ± 7%) was significantly reduced by 21.5% relative to the N2 WT worm egg hatch rate (98.5% ± 1.4%) (Figure 2E). Finally, the orientation of eggs in the hermaphrodite gonad was evaluated, because egg arrangement is also known to influence egg-laying capacity. Indeed, the *fbxl-1* adult gravid worms had abnormal egg orientation within the gonad, where the majority of egg positions were deranged with apparent migration defects, including incorrect dorsal/ventral polarity (Figure 2, F and G). Whereas the N2 WT adult worm gonads contained 1 or 2 well-aligned rows of embryos, the adult *fbxl-1(ok3741)* worms were filled with eggs, which may have resulted in unlaid embryos. Therefore, we postulate that this abnormal egg orientation underlaid the significantly increased body width observed in the *fbxl-1(ok3741)* worms. Collectively, these data demonstrate that *fbxl-1(ok3741)* worms have significantly impaired egg orientation, brood size, and hatch rate as well as delayed larval development and reduced linear growth in young adult worms.

### fbxl-1(ok3741) worms displayed globally impaired neurologic and/or muscular activity involving their pharyngeal pump rate, body-bend locomotion, and swimming activity.

**Figure 3:**
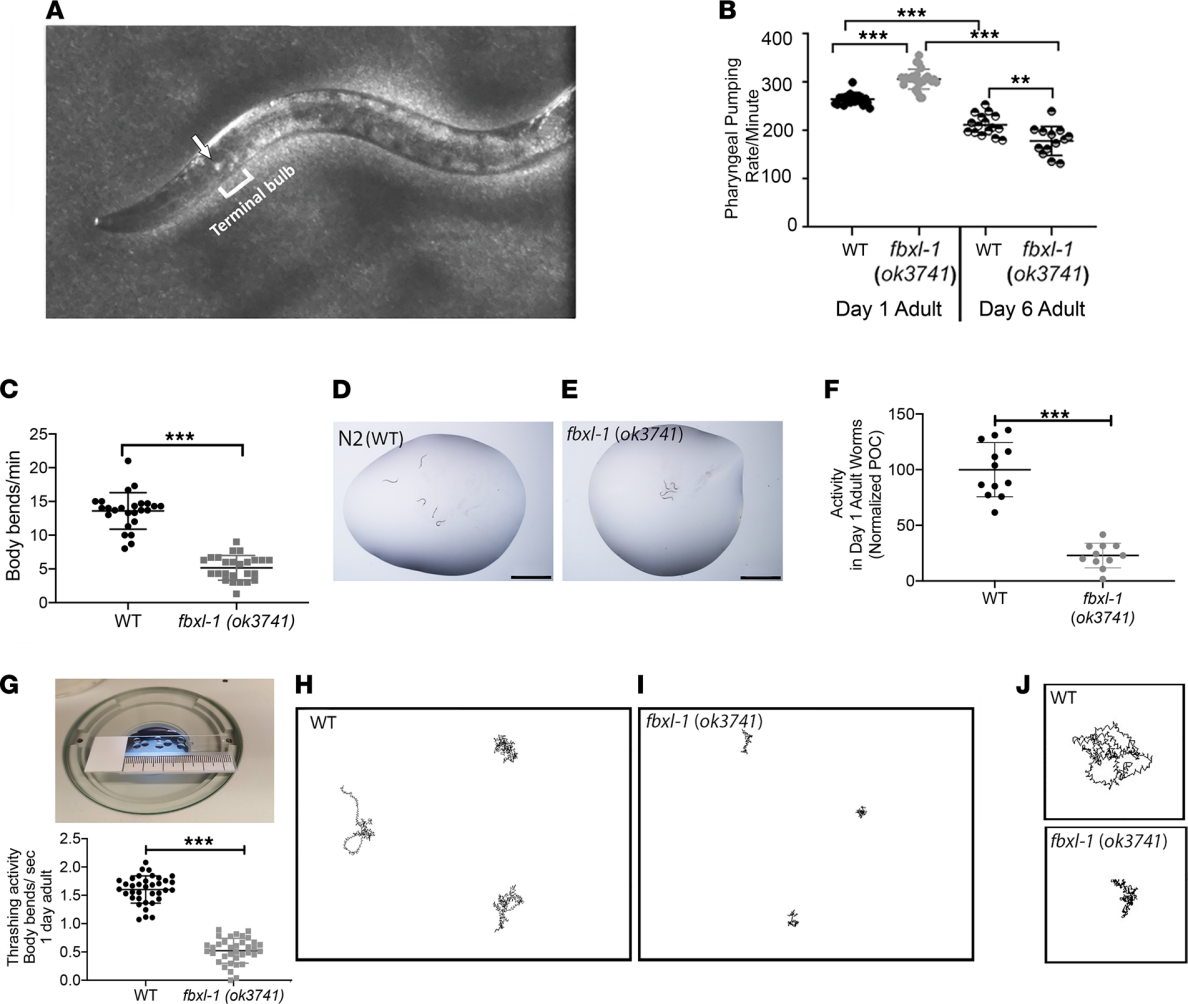
*fbxl-1*(*ok3741)* worms had impaired neuromotor function. (**A**) Pharyngeal pump rate experimental overview (Supplemental Video 1). WT *C*. *elegans* animal observed under dissecting microscope using oblique illumination; the grinder appears as a white dot (arrow) in terminal bulb. **(B**) Pharyngeal pumping rates of *fbxl-1(ok3741)* and WT worms at 1- and 6-day adult stage (*n =* 20 per strain at 1-day adult stage; *n =* 25 per strain at 6-day adult stage). Bonferroni’s correction method was applied to account for multiple comparisons, and significant findings still held. (**C**) *fbxl-1*(*ok3741)* and WT body bend rate on agar plates (Supplemental Videos 2 and 3). *n =* 25 worms per strain; 5 biological replicates performed. (**D**–**F**) Worm swimming activity analysis in liquid media. (**D** and **E**) Five worms were transferred to each drop of liquid media (scale bar: 1 mm) on a glass slide. Activity of all 5 worms in aggregate was recorded, and pixel change was analyzed using ZebraLab. (**F**) *fbxl-1*(*ok3741)* worm swimming activity was significantly lower than WT worms (Supplemental Videos 4 and 5). Each dot indicates the total activity of 5 worms in a single technical replicate. *n =* 12 for WT; *n =* 11 for *fbxl-1*(*ok3741*); with a total of 60 WT and 55 *fbxl-1*(*ok3741*) worms analyzed across 4 biological replicates. (**G**–**J**) Thrashing activity assay. (**G**) Each *fbxl-1*(*ok3741)* and WT worm was transferred to 5 μL liquid media for individual worm activity analysis by wrMTrck plugin (ImageJ). The graph shows the BBPS, which were significantly decreased in *fbxl-1*(*ok3741)* worms as compared with WT worms. Each dot indicates activity of an individual worm. *n =* 36 each strain. (**H** and **I**) Representative traces of worm movements detected by wrMtrck analysis. *fbxl-1*(*ok3741)* worms showed less activity and uncoordinated movements than WT worms. (**J**) Higher-magnification images of representative individual *fbxl-1*(*ok3741)* and WT worm traces. Data are shown as the mean ± SD. Significance was determined using unpaired Student’s *t* test. *P < 0.05, **P < 0.01, ***P < 0.001.

Human *FBXL4^–/–^* disease reduces mitochondrial content and impairs RC function, leading to diverse neurologic and muscular manifestations as well as reduced exercise capacity (2). Therefore, we performed a range of quantitative assessments to determine whether *fbxl-1(ok3741)* mutant worms recapitulated the impaired neuromuscular activity typical of *FBXL4^–/–^*-based mitochondrial disease. First, pharyngeal pump rate analysis was used to quantify integrated neurologic and/or muscular feeding activity, as the pharynx functions as a discrete neuromuscular pump that allows the ingestion of *E*. *coli* bacteria at the anterior end of the worm’s alimentary tract. Coordinated pharyngeal movements are required for efficient feeding and survival in nature, and several methods are available to quantify pharyngeal pump rate (29–33). Here, we illuminated living worms under a stereomicroscope by sliding the diaphragm to project the light obliquely onto the worm, which created a high-contrast image to allow visualization of the grinder within the terminal pharyngeal bulb as a bright spot (Figure 3A). Pharyngeal pump rate was quantified by manually counting grinder contractions in the adult worm’s head at both day 1 and day 6 in the adult stage (Figure 3A and Supplemental Video 1; supplemental material available online with this article; https://doi.org/10.1172/jci.insight.156346DS1). During pumping, the terminal pharyngeal bulb muscle contraction inverts the grinder to allow the transition of bacteria from the isthmus to the intestine, after which muscle relaxation returns the grinder to its relaxed position and allows the lumen of the corpus to close, as previously described (34). This mechanism is visible as 3 differently shaped and, in our case, bright, grinder movements (Supplemental Video 1). To estimate the pharyngeal pump rate, 1 set of 3 movements was counted as 1 pump. In day 1 adults, pharyngeal pumping showed an uncoordinated movement, with a rate that was significantly increased by 15.8% in *fbxl-1(ok3741)* worms (307 ± 21 pumps/min) as compared with N2 WT worms (265 ± 11 pumps/min) (Figure 3B). As worms aged, the pharyngeal pump rate quantified in day 6 adults showed significant reduction in both strains as compared with their rates at day 1 in adulthood, but at this stage the rate was significantly decreased by 15.5% in *fbxl-1(ok3741)* worms (179 ± 29.77 pumps/min) as compared with that in N2 WT worms (212 ± 21.37 pumps/min). Overall, these data demonstrate that the pharyngeal pump rate in *fbxl-1(ok3741)* worms was rapid but uncoordinated in young adults and that it became slower than that in controls with age, consistent with progressive neuromuscular decline.

On day 1 in the young adult stage, *fbxl-1(ok3741)* locomotion movement, as measured by body bend rate on solid media (5.16 ± 1.86 bends/min), was significantly reduced by 66% relative to that of N2 WT worms (13.60 ± 2.70 bends/min) (Figure 3C and Supplemental Videos 2 and 3). Furthermore, *fbxl-1(ok3741)* worm behavior was subjectively abnormal upon visualization during these assays (Supplemental Videos 2 and 3). Whereas N2 WT worms were observed to move constantly with only occasional arrests or backward motions without completing an entire bend, *fbxl-1(ok3741)* worm movements were persistently hesitant and frequently failed to complete a maximal body bend (Supplemental Videos 2 and 3).

*fbxl-1(ok3741)* worm swimming activity was quantified on day 1 of the adult stage in liquid media by recording movements under a dissecting microscope (Supplemental Videos 4 and 5) by a semiautomated method (ref. 25; Figure 3, D and E). Their swimming activity in liquid media was recorded for 1 minute, and the videos were analyzed using ZebraLab software (Viewpoint) after optimizing settings for both strains (25). *fbxl-1(ok3741)* worm swimming activity was decreased by 77% (23% ± 11%) as compared with N2 WT worm activity (100% ± 24%) (Figure 3F and Supplemental Videos 4 and 5). *C*. *elegans* thrashing assays were also performed to quantify body bends per second (BBPS) in liquid media by recording worm movements in liquid for 1 minute (Figure 3G) with video analysis, using the wrMTrck plugin for ImageJ (NIH) (35). Thrashing activity was significantly decreased by 67% in *fbxl-1(ok3741)* worms (0.52 ± 0.22 BBPS) as compared with N2 WT worms (1.60 ± 0.24 BBPS) (Figure 3G). Visualization of worm movements tracked by the software further substantiated that *fbxl‑1(ok3741)* worms have impaired body bend patterns, with less distance moved and lower bend amplitude, as compared with N2 WT worms (Figure 3, H–J). Collectively, 3 independent quantitative measures of locomotor activity yielded a similar estimate of approximately 70% (range 66%–77%) reduced locomotor activity in *fbxl‑1(ok3741)* worms (Figure 3). Overall, *fbxl-1(ok3741)* worms displayed globally impaired neurologic and/or muscular activity involving their pharyngeal pump rate, body-bend locomotion, and swimming activity.

### DCA treatment rescued fecundity, pharyngeal pumping function, and biochemical deficiencies at the level of RC enzyme activity and intracellular lactate in fbxl-1(ok3741) worms.

**Figure 4:**
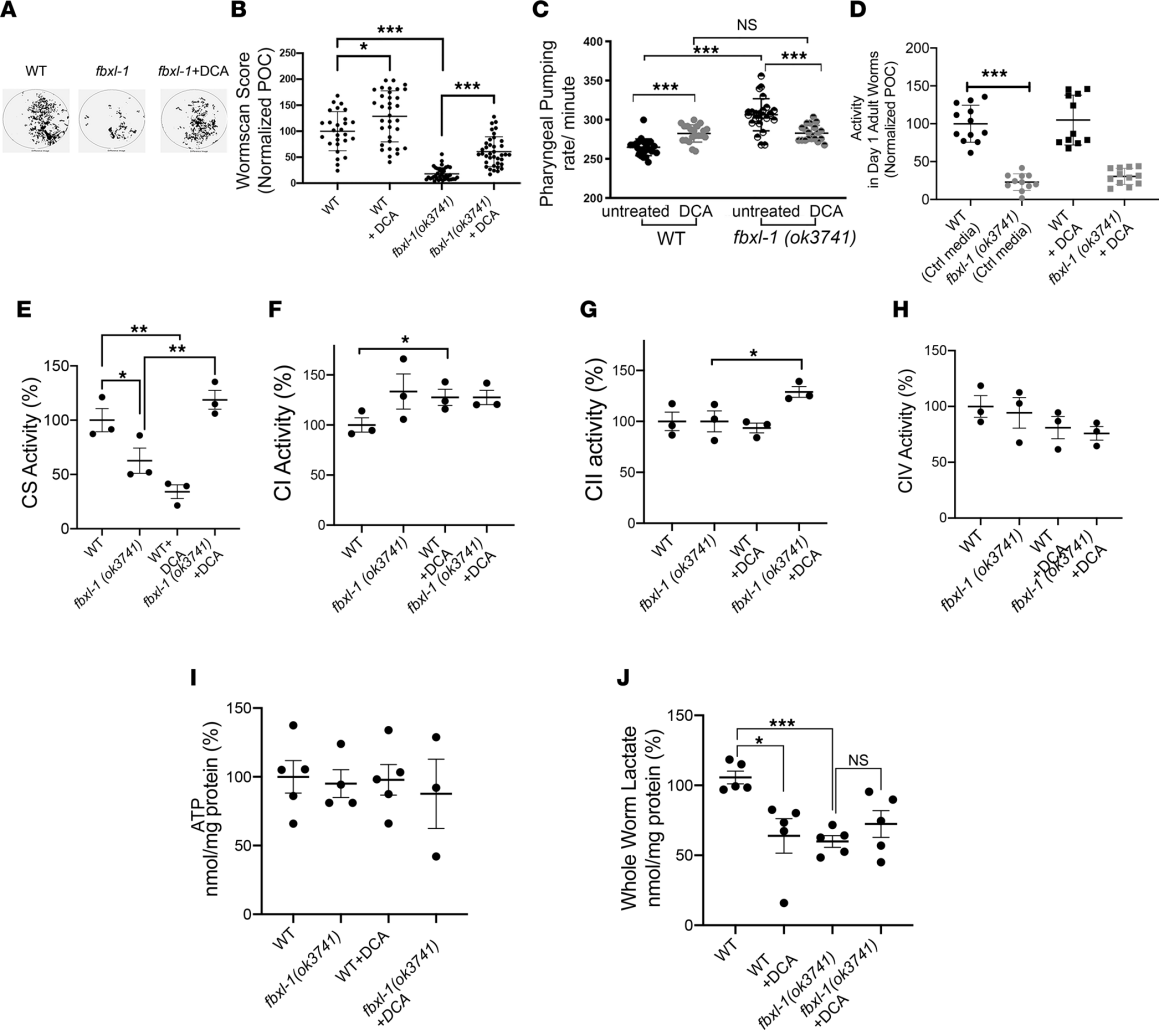
DCA treatment rescued egg-laying behavior, pharyngeal pumping function, and mitochondrial function in *fbxl-1(ok3741)* worms. (**A**) Visualization of worm progeny using semiautomated Wormscan method, showing reduced density of *fbxl-1(ok3741)* worms as compared with WT worms in control media, and recovered density of signal in the well with DCA-treated *fbxl-1(ok3741)* worms. (**B**) Difference image score obtained with WormScan method and normalized using percentage of control (POC); *fbxl-1(ok3741)* worms (*n =* 38) and WT worms at baseline (*n =* 35); *n* = 57 for 25 mM DCA treated *bxl-1(ok3741)* and *n* = 31 for DCA-treated WT worms; (mean ± SEM). (**C**) Pharyngeal pump rate in untreated and DCA-treated (25 mM) 1-day adult *fbxl-1*(*ok3741*) and N2 WT worms. *n =* 25 worms/strain in control media; *n =* 20 worms/strain in DCA media; 3 biological replicates. (**D**) Untreated and DCA-treated worms’ swim activity analyzed by ZebraLab; *n* = 12 DCA-treated worms; *n* = 11 untreated worms; (mean ± SD). (**E**–**H**) RC enzyme activity of CS, complex I (CI), CII, and CIV was quantified in untreated and DCA-treated WT and *fbxl- 1(ok3741)* synchronized day-1 adult worm populations. All rates are expressed as percentage of normalized WT control. *n* = 3 per condition; (mean ± SEM). One-tailed *t* test performed for statistical analysis. (**I**) ATP levels in whole worms were unchanged in *fbxl-1(ok3741)* worms relative to WT worms in untreated and DCA-treated conditions (mean ± SEM). *n* = 5 each strain. (**J**) Whole worm lactate evaluated in *fbxl-1(ok3741)* worms relative to WT worms (mean ± SEM). *n* = 5 each strain. Significance was determined using unpaired Student’s *t* test. *P < 0.05, **P < 0.01, ***P < 0.001. Bonferroni’s correction method was applied to account for multiple comparisons, and significant findings still held.

Given that *C*. *elegans* is a robust translational model in which to not only dissect disease mechanisms, but also to evaluate candidate therapies for mitochondrial disease (24, 36), we sought to evaluate whether the significantly impaired fecundity, development, growth, and neurologic and/or muscular activity of *fbxl-1(ok3741)* worms could be rescued with empiric mitochondrial disease therapies (37). Specifically, 12 drugs that have been empirically used or postulated as candidate human RC disease therapies (24, 38–42) were screened for their ability to rescue the significantly reduced the brood size of *fbxl-1(ok3741) worms* (Supplemental Table 1 and data not shown). Only DCA substantially improved *fbxl-1(ok3741)* worms’ reduced brood size. WormScan analysis was performed to validate DCA effects on fecundity by quantifying an integrated measure of brood size, egg hatch rate, and larval developmental in a 96-well plate format (43). Specifically, 2 L4 stage worms per well were grown in S basal media (5.85 g NaCl, 1 g K2 HPO4, 6 g KH2PO4, 1 ml cholesterol [5 mg/ml in ethanol], H2O to 1 liter) with OP50 *E*. *coli* for 4 days with either buffer control or DCA treatment (Figure 4A). WormScan results validated those seen by manual brood size analysis (Figure 2D). *fbxl-1(ok3741)* fecundity (19% ± 2% of normalized control, mean ± SEM) was significantly reduced by 81% relative to N2 WT worms *(*100% ± 7% of normalized control) (Figure 4, A and B). DCA (25 mM) significantly rescued *fbxl-1(ok3741)* fecundity by 2.9-fold in biological replicate experiments, with 74% ± 6.5% normalized to controls in DCA-treated *fbxl-1(ok3741)* worms as compared with 19% ± 2% in buffer-only exposed *fbxl-1(ok3741)* worms (Figure 4B). Interestingly, DCA treatment also significantly improved, although to a lesser extent, the fecundity by 25% percent of that of N2 WT worms (Figure 4B). Thus, despite the high molar concentration of DCA fed to *C*. *elegans*, no toxic effects were seen on L4 worm development or adult worm activity. Indeed, DCA significantly improved the grossly impaired fecundity of *fbxl-1(ok3741)* mutants as well as WT animals.

DCA treatment was further studied to evaluate whether it rescued the impaired neurologic and/or muscular activity of the *fbxl-1(ok3741)* worms at the level of pharyngeal pump rate and swimming activity. Specifically, pharyngeal pump rate was quantified in day 1 adult stage N2 WT and *fbxl-1(ok3741)* worms exposed from the egg stage on NGM plates to 25 mM DCA in S. basal media spread with *OP50 E*. *coli* on a 25 mL agar plate. The abnormally increased pharyngeal pump rate of day 1 adult *fbxl-1(ok3741)* worms was significantly reduced with DCA treatment by 23% (283 ± 10 pumps/min) relative to that of untreated *fbxl-1(ok3741)* worms (307 ± 21 pumps/min) (Figure 4C); this effect normalized the pharyngeal pump rate so it was comparable to that observed in DCA-treated N2 WT worms (283 ± 11 pumps/min), as DCA treatment significantly increased the N2 WT pharyngeal pump rate by 27% percent over that of N2 WT baseline (Figure 4C). The ability of DCA to rescue *fbxl-1(ok3741)* worms’ reduced swimming activity was studied using a ZebraLab software analytic method (25), but no significant difference was detected in the treated mutant worms (30% ± 11%) as compared with the untreated ones (23% ± 11%) (Figure 4D). Thus, DCA rescued neurologic and/or muscular activity at the level of pharyngeal pump rate but did not improve swimming activity and developmental delay (data not shown).

Given the significant improvements in gross phenotypes at the level of fecundity and pharyngeal pump rate of *fbxl-1(ok3741)* worms that occurred upon treatment with DCA, we sought to determine whether DCA improved the mitochondrial dysfunction and variably reduced mitochondrial RC enzyme activities typical of *FBXL4* disease (2). Indeed, *fbxl-1(ok3741)* worm citrate synthase (CS) activity was reduced to 42% ± 8% (mean ± SEM) relative to that of N2 WT worms (Figure 4E). mtDNA level was also measured, but it was unchanged in the mutant strain compared with the N2 WT strain (Supplemental Figure 2; ref. 44). Importantly, DCA treatment from egg stage through day 1 adult stage significantly increased relative CS activity by 47% in *fbxl-1(ok3741)* worms, from 42% ± 8% in untreated animals to 79% ± 6% in DCA-treated animals (Figure 4E). RC complex I, II, and IV enzyme activities were not significantly different in *fbxl-1*(*ok3741*) mitochondria as compared with N2 WT mitochondria (Figure 4, F–H). Nor were whole worm ATP levels significantly altered in *fbxl-1(ok3741)* worms (Figure 4I). Nonetheless, DCA treatment increased mitochondrial complex II enzymatic activity (Figure 4G) in *fbxl-1(ok3741)* worms (129% ± 5.22%), which is suggestive of a mitochondrial proliferative response, as complex II comprises only nuclear-encoded subunits that increase directly with mitochondrial content. DCA also increased complex I activity in the N2 WT worms (127.6% ± 8.1%) (Figure 4F). Surprisingly, tissue lactate levels were significantly decreased in day 1 adult stage untreated *fbxl-1(ok3741)* worm homogenate by 40% as compared with N2 WT worms (Figure 4J). DCA treatment significantly decreased intracellular lactate levels of N2 WT worms (64% ± 12%) relative to untreated N2 WT animals. By contrast, DCA treatment of *fbxl‑1*(*ok4741*) worms led to a nonsignificant trend toward increased intracellular lactate levels (73.4% ± 9.6%) relative to untreated *fbxl‑1*(*ok4741*) worms (Figure 4J). Overall, these data demonstrate that *fbxl-1(ok3741)* worms have significant mitochondrial dysfunction and decreased intracellular lactate levels without alteration in mitochondrial RC enzyme activities or ATP levels. Interestingly, DCA significantly rescued the activity of CS and trended toward normalization of intracellular lactate levels in the *fbxl-1(ok3741)* worms.

### fbxl4^sa12470^ zebrafish larvae showed liver stenosis and mitochondria ultrastructural damage.

**Figure 5:**
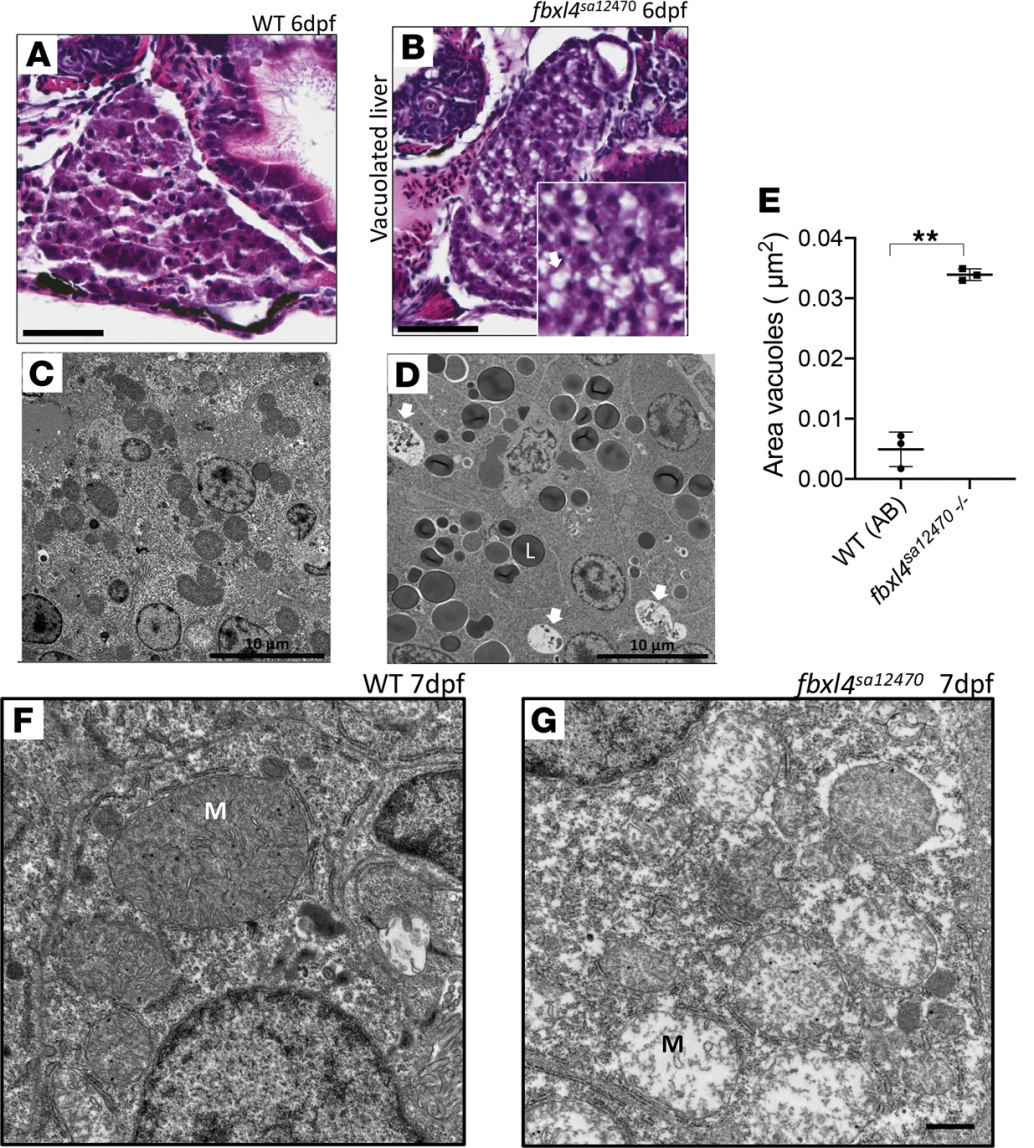
Biochemical analysis, histopathology, and ultrastructural investigation of homozygous *fbxl4*^sa12470^ zebrafish liver. (**A**) Liver histology in AB (WT) zebrafish larvae at 6 dpf. Scale bar: 25 μm. (**B**) Vacuolated liver in homozygous *fbxl4*^sa12470^ zebrafish larvae at 6 dpf. 100% of larvae (*n =* 25) showed vacuolated liver, while no WT larvae (*n =* 12) showed the disease phenotype. Scale bar: 25 μm. (**C**) Ultrastructure of hepatocytes in 7 WT dpf zebrafish larvae showing normal ultrastructure. Scale bar: 10 μm. (**D**) Ultrastructure of hepatocytes in mutant 7 dpf zebrafish larvae showing increased rate of lipid droplets (L) and autophagic vacuoles (white arrows). Scale bar: 10 μm. (**E**) Total area of vacuoles occupying liver areas estimated in μm^2^ in mutant versus WT larvae at 7 dpf. Total area of liver analyzed: 1,978 μm^2^ (WT) and 2,741 μm^2^ (*fbxl4^sa12470^*). **P < 0.01. Significance was determined using unpaired *t* test. *n =* 3 each fish line. (**F**) Mitochondrial ultrastructure (M) in hepatocytes of 7 dpf WT zebrafish larvae. (**G**) Loss of normal matrix electron density and mitochondrial cristae damage (M) was observed in hepatocytes of 7 dpf *fbxl4^sa12470–/–^* larvae. Scale bar: 1 μm (**F** and **G**).

*fbxl4^sa12470^* larvae did not show any gross morphological phenotype when observed under a dissecting microscope, but histopathology showed an increased rate of vacuolated liver indicative of stenosis, in 6 dpf homozygous larvae, with 100% of homozygous larvae showing the disease liver phenotype, whereas no WT larvae showed vacuolated liver (Figure 5, A and B). This phenotype may indicate an increased fat level in the homozygous liver. Indeed, ultrastructural analysis of 6 dpf homozygous larvae showed an increased number of large lipid droplets and a significantly increased area of autophagic vacuoles (mutant, 0.034 ± 0.0009 versus WT, 0.005 ± 0.0028 μm^2^; mean ± SD; Figure 5, C–E). Ultrastructural analysis of 7 dpf homozygous larvae also showed mitochondrial ultrastructural damage, with loss of normal matrix electron density and mitochondrial cristae impairment (Figure 5, F and G; statistical analysis was not performed).

### Cross-evolutionary species validation studies demonstrated that DCA treatment prevented brain death and neurologic and/or muscular dysfunction in fbxl4^sa12470^ zebrafish larvae exposed to CAP.

**Figure 6:**
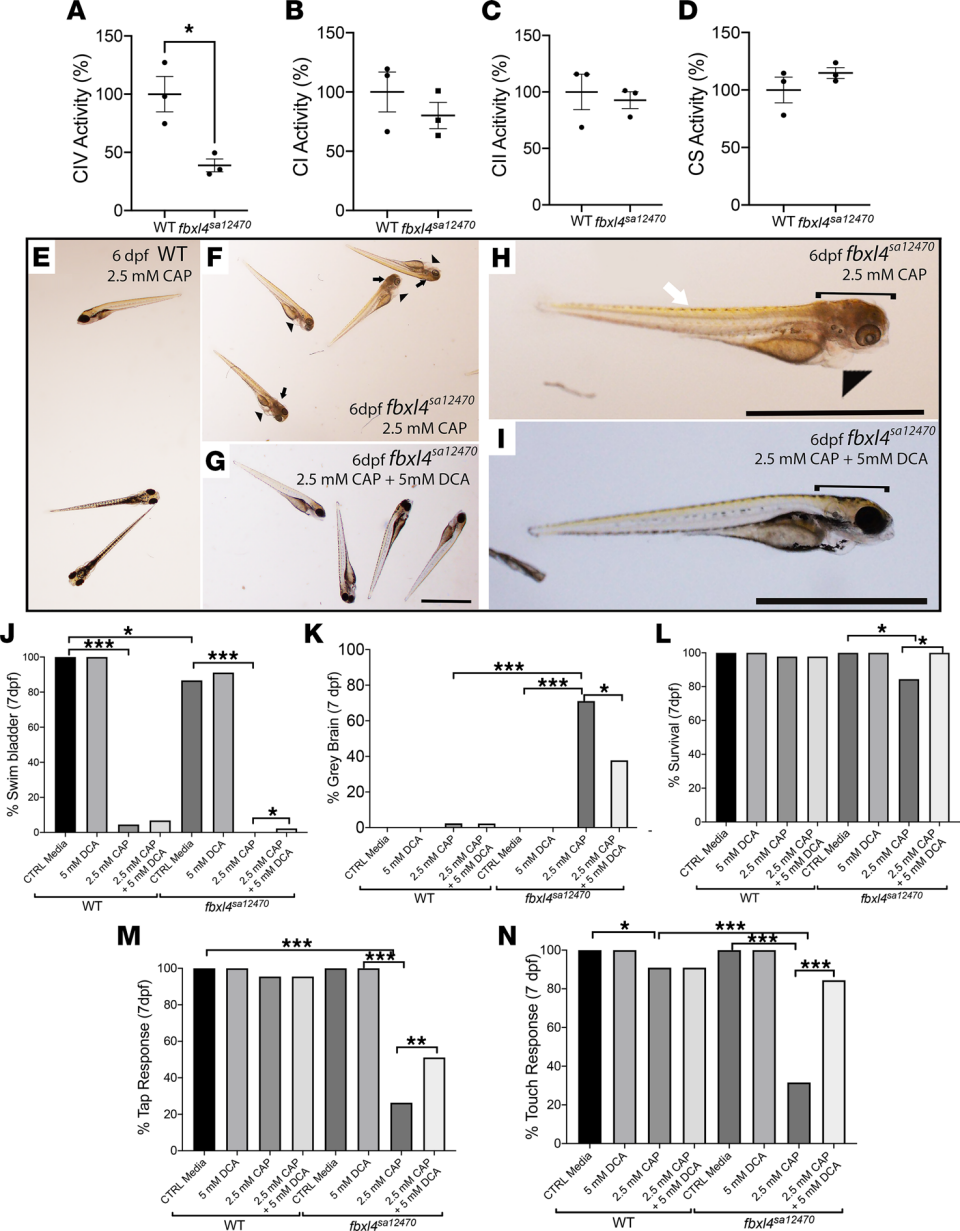
DCA rescued the gray brain phenotype, survival, and integrated neurologic and/or muscular function in CAP-stressed *fbxl4^sa12470^* larval zebrafish. (**A**–**D**) RC and CS enzyme activity detected in 7 dpf larvae (mean ± SEM). *n =* 3 each condition. (**E**–**I**) Representative images of 6 dpf age-matched *fbxl4^sa12470^* and AB WT larvae at 6 dpf after 4 days (starting from 2 dpf) of incubation with 2.5 mM CAP alone or with DCA cotreatment. Morphological defects were obvious at 6 dpf, showing higher sensitivity of (**F** and **H**) *fbxl4^sa12470^* larvae to 2.5 mM CAP compared with (**E**) AB WT larvae: gray brain phenotype (black arrows in **F** and bracket in **H**, Supplemental Figure 3), heart edema (generally not observed in WT, arrowheads in **F** and **H**), and overall body degeneration and slight bent tail (white arrow in **H**). (**G** and **I**) Coexposure of stressed *fbxl4*^sa12470^ larvae with 5 mM DCA improved the gray brain phenotype (clear brain in **I** indicated by a bracket; Supplemental Figure 3) but did not rescue the delay in swim bladder formation in either AB WT or *fbxl4^sa12470^* larvae. Scale bar: 1 mm. (**J**–**N**) 2.5 mM CAP significantly affected development (percentage of swim bladder), survival, and neuromuscular response (percentage of tap response and touch response), and caused brain death in 7 dpf mutant larvae (Supplemental Table 2, A and B). (**J** and **K** and Supplemental Videos 6 and 7) 5 mM DCA significantly rescued the gray brain phenotype and survival and improved neuromuscular response. *P < 0.05, **P < 0.01, ***P < 0.001, Cochran-Mantel-Haenszel and χ^2^ test performed (Supplemental Table 2). Bar graphs are representative of the statistical analysis shown in the Supplemental Table 2, where all data details are shown.

*fbxl4^sa12470^* larvae grown under basal conditions showed a significant decrease in complex IV enzyme activity (39% ± 5% vs. 100% ± 15% in WT larvae, Figure 6A), while CI, CII, and CS activities were unchanged compared with WT larvae (Figure 6, B–D). No gross morphological defects were evident upon dissecting microscope visualization. Therefore, we evaluated the phenotypic effects of exposing *fbxl4^sa12470^* zebrafish larvae to a mitochondrial translation inhibitor, CAP, as *FBXL4* disease can impair multiple RC complex enzyme activities in different tissues (2), and 3 mM CAP was previously shown to decrease cell viability in primary fibroblast cell lines from *FBXL4^–/–^* disease patients (24). Indeed, *fbxl4^sa12470^* larvae were hypersensitive to CAP-based pharmacologic inhibition of the RC, similar to findings in our previous reported for AB WT zebrafish larvae (23), with significantly decreased survival and RC complexes I and IV enzyme activities. Morphological defects were seen with increased frequency by 6 dpf as compared with AB WT fish when *fbxl4^sa12470^* larvae were exposed to 2.5 mM CAP in control media from 2 dpf, including heart edema (generally not observed in AB WT fish), slight bent tail (45), organ degeneration, and gray brain, which is indicative of brain degeneration and atrophy, as previously described in Byrnes et al. (23), Polyak et al. (46), Guha et al. (24), and Guha et al. (39) (Figure 6, E–I, and Supplemental Figure 3).

Coexposure of *fbxl4^sa12470^* larvae from 2 dpf to 2.5 mM CAP and 5 mM DCA substantially decreased overall morphological abnormalities, including edema, bent tail, and the gray brain phenotype suggestive of brain death (Figure 6, G and I) compared with buffer-only CAP-exposed larvae (Figure 6, F and I). However, DCA treatment did not rescue the developmental delay of *fbxl4^sa12470^* larvae, as assessed at 6 dpf by the absence of a swim bladder (Figure 6, G and I).

Zebrafish larval morphology and locomotor response after exposure to 2.5 mM CAP alone or with cotreatment of 5 mM DCA were statistically analyzed at 7 dpf. For morphological analyses, gross phenotype percentages were analyzed, including whether they had a swim bladder (to evaluate developmental delay, as swim bladder formation and inflation occur by 5 dpf), gray brain, and animal death (as determined by absence of a heartbeat) (Figure 6, J–L). The animal percentage with intact tap and touch responses was also assessed (Figure 6, M and N). Morphological defects after incubation in 2.5 mM CAP became obvious at 6 dpf in *fbxl4^sa12470^* larvae, reflecting hypersensitivity to 2.5 mM CAP that induced developmental delays in both WT and *fbxl4^sa12470^* larvae, without significant rescue by coexposure to 5 mM DCA. Specifically, the swim bladder was detected by 7 dpf in only 4.5% of AB WT and 0% of *fbxl4^sa12470^* larvae after incubation in 2.5 mM CAP alone and in 6.8% of AB WT and 2.2% of *fbxl4^sa12470^* larvae after cotreatment with 5 mM DCA (Figure 6J and Supplemental Table 2, A and B). DCA significantly rescued the gray brain phenotype in CAP-exposed *fbxl4^sa12470^* larvae. Specifically, 2.5 mM CAP exposure from 2 dpf significantly increased gray brain induction in *fbxl4^sa12470^* larvae (71.1%) as compared with AB WT larvae (2.4%); this was significantly prevented upon coexposure of 7 dpf *fbxl4^sa12470^* larvae to 5 mM DCA (37.8%), without any effects on AB WT larvae (2.3%) (Figure 6K and Supplemental Table 2, A and B). Interestingly, while 2.5 mM CAP strongly impaired mutant fish morphology at the level of swim bladder formation and the gray brain phenotype, suggestive of gross brain death, it only mildly decreased *fbxl4^sa12470^* larvae survival, as evaluated by the presence of a heartbeat at 7 dpf. Zebrafish larvae that showed the gray brain phenotype were not able to move and/ or respond to any stimulus, and this phenotype was followed shortly thereafter by organismal death. Specifically, *fbxl4^sa12470^* and AB WT larval survival after incubation in 2.5 mM CAP decreased by 15.6% and 2.2%, respectively. However, survival was fully normalized in *fbxl4^sa12470^* larvae with DCA treatment (100% present heartbeat) (Figure 6L). Finally, *fbxl4^sa12470^* larval neurologic and muscular response were significantly decreased by exposure to 2.5 mM CAP, with only minimal effects in AB WT larvae. Specifically, only 26.3% and 31.6% of *fbxl4^sa12470^* larvae displayed tap- and touch-evoked responses, respectively, compared with 96.0% and 90.9% of AB WT zebrafish larvae (Figure 6, M and N, and Supplemental Table 2, A and B). These locomotor responses were significantly increased to 51.1% and 84.4% in *fbxl4^sa12470^* larvae after coexposure with 5 mM DCA (*P <* 0.001 compared with AB WT) (Figure 6, M and N; Supplemental Table 2, A and B; and Supplemental Videos 6 and 7). Tap- and touch-evoked responses did not vary with DCA treatment in AB WT zebrafish larvae. Overall, these data demonstrate that *fbxl4^sa12470^* larvae exposure to the mitochondrial translation inhibitor CAP results in developmental delay, neurologic and/or muscular dysfunction, brain death, and reduced survival. DCA cotreatment during the exposure to CAP significantly prevented or ameliorated all of these abnormalities in *fbxl4^sa12470^* larvae, with the exception of swim bladder formation.

### DCA treatment rescued mitochondrial dysfunction in FBXL4^–/–^ human fibroblasts.

**Figure 7:**
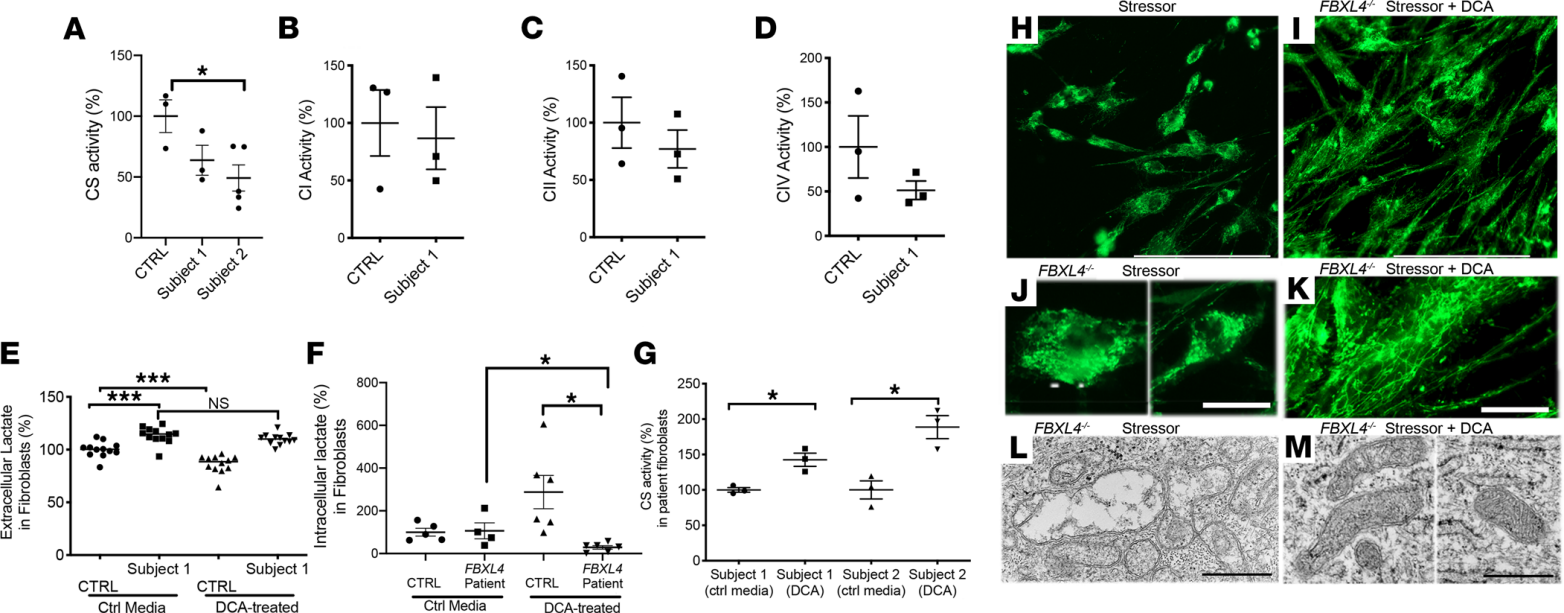
*FBXL4^–/–^* human fibroblasts had reduced CS activity and abnormal mitochondrial structure that was improved with DCA treatment. (**A**) CS activity decreased in patient fibroblasts from participants 1 (*n =* 3) and 2 (*n =* 5) as compared with control fibroblasts (*n =* 3). (**B**–**D**) RC enzyme activity was not significantly changed in patient fibroblasts compared with control fibroblasts. (**E**) Extracellular lactate levels were significantly decreased in patient fibroblasts compared with control cells in control media. DCA treatment did not affect extracellular lactate levels in patient cells. *n =* 12 each condition. (**F**) Intracellular lactate level evaluation in untreated and DCA-treated (20 mM) fibroblasts from participant 1 and controls. *n =* 12 each condition. (**G**) CS activity after treatment of fibroblasts with 20 mM DCA (participants 1 and 2). CS activity level in treated fibroblasts was normalized for percentage in control media. Data are shown as the mean ± SEM. Significance was determined using unpaired Student’s *t* test. *P < 0.05, ***P < 0.001. Bonferroni’s correction method was applied for **A** and **E**–**G** to account for multiple comparisons, and significant findings still held. (**H** and **I**) Low-magnification images (original magnification, ×20) of *FBXL4^–/–^* fibroblasts from participant 1 after exposure to metabolic stressor and cotreatment with 20 mM DCA for 48 hours. Mitochondria were stained with MitoTracker green (Thermofisher Scientific). Scale bar: 200 μm. (**J** and **K**) High-magnification images of mitochondrial morphology in *FBXL4^–/–^* fibroblasts from participant 1 after exposure to metabolic stressor and cotreatment with 20 mM DCA for 48 hours (see representative fibroblast image from participant 2 in Supplemental Figure 5). Mitochondria (in green) were disarranged and fragmented when incubated with stressor. More elongated mitochondria were visible after coincubation with 20 mM DCA. Scale bar: 25 μm. (**L** and **M**) EM images of mitochondria in *FBXL4^–/–^* fibroblasts from participant 1, showing loss of matrix electron density and loss of mitochondrial cristae after incubation in metabolic stressor for 48 hours and rescue of mitochondrial ultrastructure, with defined cristae and normal matrix electron density after cotreatment with 20 mM DCA. Scale bar: 500 nm.

Consistent with *FBXL4^–/–^* disease causing variable degrees of mitochondrial dysfunction, CS activity, used as a proxy for mitochondrial content, showed a trend toward decrease by 51% in fibroblasts from participant 1 (49% ± 12%, mean ± SEM) and was significantly decreased by 56% in fibroblasts from participant 2 (44 ± 11%) when normalized to healthy control human fibroblasts (100% ± 13%) (Figure 7A). RC enzyme activity analysis performed in participant 1 showed a trend toward lower complex IV activity that was not significantly different relative to that of healthy human controls (Figure 7D). Specifically, RC complex I, II, and IV enzymatic activities in participant 1 fibroblasts were 87% ± 27%, 77% ± 16%, and 51% ± 10%, respectively, compared with control fibroblasts (Figure 7, B–D). Thus, *FBXL4^–/–^* disease fibroblasts manifest significant impairment of CS activity and a nonsignificant trend toward complex IV deficiency.

*FBXL4^–/–^* disease is also often associated with lactic acidemia, detectable in blood or plasma. Therefore, we evaluated both extracellular and intracellular lactate levels in *FBXL4^–/–^* disease fibroblasts relative to controls. Indeed, significantly increased extracellular lactate was seen in fibroblasts from participant 1 (113% ± 2%; mean ± SEM) normalized to healthy controls (Figure 7E). In contrast, the intracellular lactate level was unchanged in fibroblasts from participant 1 (106% ± 37%; mean ± SEM) as compared with controls under basal conditions (Figure 7F). Thus, *FBXL4^–/–^* disease fibroblasts manifest increased extracellular, but not intracellular, lactate levels.

DCA (20 mM) treatment effects for 48 hours on both mitochondrial content and lactate levels were assessed in *FBXL4^–/–^* human fibroblasts. Interestingly, DCA treatment did not alter the increased extracellular lactate levels of *FBXL4^–/–^* fibroblasts but did significantly decrease extracellular lactate in healthy control fibroblasts (Figure 6E). Intracellular lactate was reduced after DCA treatment in fibroblasts from participant 1 (29% ± 8%, mean ± SEM) as compared with untreated fibroblasts (106% ± 37%). A significant difference was also observed between the DCA-treated WT fibroblasts and DCA-treated fibroblasts from participant 1. (Figure 7F). CS activity was significantly increased by DCA treatment in both *FBXL4^–/–^* participant fibroblasts by 30% and 47% relative to healthy control fibroblasts in control media (142% ± 9% in participant 1 and 189% ± 28% in participant 2; mean ± SEM; Figure 7G), but it was not altered by DCA treatment in healthy control fibroblasts (data not shown). Collectively, these preclinical data in human *FBXL4^–/–^* fibroblasts support the therapeutic potential of DCA for ameliorating mitochondrial biochemical dysfunction, despite having a clear benefit on extracellular or intracellular lactate levels.

*FBXL4^–/–^* disease fibroblasts from participant 1 also manifested mitochondrial damage; this was on the basis of qualitative analysis of their mitochondrial morphology and ultrastructure following incubation under conditions of metabolic stress invoked by growth in DMEM media without glucose and uridine. Indeed, cellular damage with mitochondrial fragmentation was observed only in *FBXL4^–/–^* human fibroblasts (Figure 7, H–K) but not in healthy control fibroblasts under these growth conditions (Supplemental Figure 4). DCA (20 mM) cotreatment for 48 hours rescued the mitochondrial morphology of *FBXL4^–/–^* human fibroblasts, specifically reducing mitochondrial fragmentation, as was visualized by more elongated and filamentous mitochondria (Figure 7K, see also Supplemental Figure 5). Ultrastructural analysis by electron microscopy also clearly demonstrated damaged mitochondria with loss of matrix electron density and cristae in *FBXL4^–/–^* human fibroblasts incubated under the metabolic stress conditions for 48 hours; the apparent damage was ameliorated by 20 mM DCA treatment (Figure 7, L and M). Thus, DCA treatment rescued both gross mitochondrial morphology and ultrastructural abnormalities that occurred under growth conditions of metabolic stress.

## Discussion

*FBXL4^–/–^* disease is a severe, multisystemic mitochondrial disorder involving mitochondrial depletion and RC complex deficiencies with lactic acidemia; no FDA-approved effective therapies exist for this disease. Here, we characterized what we believe to be novel genetic models of *FBXL4^–/–^* mitochondrial disease and evaluated the preclinical efficacy of DCA as a candidate therapy across 3 evolutionarily distinct model species, namely *C*. *elegans*, zebrafish, and human fibroblasts.

Indeed, developmental delay, abnormal growth, reduced fecundity, impaired neurologic and/or muscular and locomotor activity, mitochondrial dysfunction, and altered lactate metabolism were identified in *fbxl-1(ok3741) C*. *elegans*. The *fbxl4^sa12470^* zebrafish larvae showed decreased CIV enzyme activity, liver histopathology, and mitochondrial ultrastructural damage at the baseline. Screening of 12 empiric or predicted candidate drugs was performed by brood size analysis in the *C*. *elegans* mutant*,* with significant rescue seen only with DCA, a PDHc activator. Detailed studies of DCA effects in *fbxl-1(ok3741)* *C*. *elegans* demonstrated that it rescued fecundity, neurologic and/or muscular activity, and biochemical deficiencies involving RC enzyme activities. Validation studies were extended to a vertebrate model of *FBXL4^–/–^* disease, namely *fbxl4^sa12470^* zebrafish larvae exposed to mitochondrial translation inhibition with CAP. In the homozygous *fbxl4^sa12470^* disease larval zebrafish model exposed to acute metabolic stress, DCA treatment significantly improved survival and prevented neurologic and/or muscular dysfunction that correlated with their gray brain phenotype. Indeed, this acute brain-graying phenotype was suggestive, at the gross level, of brain death, as has been seen similarly across a range of mitochondrial RC pharmacologic inhibitor and/or genetic zebrafish models of Leigh syndrome spectrum disorders, in which direct impairment of RC function consistently caused pronounced graying of the brain that immediately preceded an animal’s loss of neurologic and/or muscular touch and startle responses, followed shortly thereafter by organismal death, as evidenced by loss of heart beat (23, 24, 39, 46). The exact cellular mechanism(s) of brain death remain to be identified. Further validation studies performed in human *FBXL4^–/–^* fibroblasts demonstrated that DCA treatment significantly rescued their mitochondrial CS activity and stress-induced morphologic and ultrastructural mitochondrial defects. Collectively, these data demonstrate that *fbxl-1*(*ok3741*) *C*. *elegans* and *fbxl4^sa12470^* zebrafish are robust translational models in which to study mechanisms and to identify preclinical therapeutic candidates for *FBXL4^–/–^* disease. Furthermore, DCA is a lead therapeutic candidate with quantifiable beneficial effects on survival, neurologic and/or muscular function, and mitochondrial physiology that now warrant rigorous clinical trial study in humans with *FBXL4^–/–^* disease.

Neuromuscular dysfunction is a hallmark symptom of human *FBXL4^–/–^* mitochondrial disease (2, 4). We identified similar grossly impaired neurologic and/or muscular functions at the levels of egg-laying behavior, locomotor activity, and pharyngeal pumping in *fbxl-1(ok3741) C*. *elegans*. Egg-laying capacity in *C*. *elegans* relies on the coordination of muscles and motor neurons to regulate vulva contraction and relaxation (47). Another key contributing factor to the decreased numbers of progeny in *fbxl-1*(*ok3741*) worms was unlaid embryos and/or reduced brood size (47), resulting from severely disarranged egg orientation in their gonad (48). *fbxl-1*(*ok3741*) *C*. *elegans* also had severely reduced locomotor activity, a phenotype previously observed in other *C*. *elegans* models of mitochondrial disease (49–51). As worm survival is dependent upon maintaining a proper pharyngeal pumping rate, the abnormal pharyngeal pumping rate observed in *fbxl-1(ok3741)* worms indicates severe myopathy and muscle weakness (52). Similarly, patients with *FBXL4^–/–^* disease have globally impaired motor skills (4, 5). Furthermore, *fbxl-1(ok3741)* 1-day adult worms display a defective contraction-relaxation cycle that indicates unsynchronized movements of the pharyngeal pump. Ultimately, pharyngeal pumping rates decrease with age in the *fbxl-1(ok3741)* worms, confirming that the severity of their impaired neurologic and/or muscular function extends to basic survival functions, such as feeding.

Mitochondrial amount and biochemical function were evaluated by CS and RC enzyme activities analyses, respectively, which showed significantly decreased CS activity in *fbxl-1*(*ok3741*) *C*. *elegans*. CS is the enzyme responsible for catalyzing the first reaction of the TCA cycle (53); it is widely considered to be a clinical biomarker of mitochondrial amount (54). Decreased CS activity may reflect abnormal mitochondrial dynamics at the level of fusion and fission necessary for mitochondrial maintenance and/or increased mitophagy (55). One possible hypothesis is that the decreased CS activity in the *fbxl-1* *C*. *elegans* and patient fibroblasts may result from increased mitochondrial fragmentation, with decreased mitochondrial mass. Indeed, a fragmented mitochondrial network with overall reduced mitochondrial content has been previously reported to occur in *FBXL4-*deficient cells (1–3), and mitochondrial fragmentation with decreased CS activity but increased mitochondrial, mass as detected by flow cytometry, was observed in fibroblasts from some patients (55). Interestingly, a recent study demonstrated that the FBXL4 protein may indeed play a role in regulating mitochondrial fusion (56) and similarly observed that *FBXL4* pathogenic variants in human fibroblasts may cause increased mitochondrial fragmentation that over time lead to reduced mitochondrial content. Overall, we validated that *FBXL4*^–/–^ human fibroblasts have reduced CS enzyme activity at baseline, similar to that seen in *fbxl-1*^–/–^ worms. Furthermore, human *FBXL4*^–/–^ fibroblast growth under metabolic stress conditions induced mitochondrial fragmentation, with ultrastructural evidence of mitochondrial matrix and cristae alterations. Most importantly, all of these alterations in mitochondrial amount and morphology were rescued in both *C*. *elegans* and human fibroblast models of *FBXL4^–/–^* disease upon treatment with DCA.

While mtDNA depletion was previously confirmed in fibroblasts from participant 1 (participant 5 in Gai, et al. 2013; ref. 2), mtDNA level was not affected in *fbxl-1*(*ok3741*) worms, at least when studied at the L4 developmental stage. However, mtDNA levels can be tissue specific (55) or vary over time due to progressive dysfunction of mitochondrial replication or mitophagy. Further investigation will be needed to assess mtDNA levels over time in both the *fbxl-1^–/–^* worm and *fbxl4^sa12470^* zebrafish models.

Surprisingly, whole worm tissue lactate levels were significantly decreased in *fbxl-1*(*ok3741*) worms relative to those in N2 WT controls. Similar to our data, however, decreased CS activity, mitochondrial ultrastructural alterations, and decreased intracellular lactate levels were also previously observed in *C*. *elegans* stressed with a mitochondrial prooxidant and complex II inhibitor, paraquat (57). Indeed, elevated lactate levels in blood from patients with *FBXL4^–/–^* disease are a commonly observed indicator of extracellular lactate accumulation (2, 4), clinically described as lactic acidemia and, when at sufficient levels to reduce blood pH, the cause of lactic acidosis. Indeed, while we observed no difference in intracellular lactate levels between *FBXL4^–/–^* disease and healthy control human fibroblast cell pellets, lactate levels collected over 48 hours in the fibroblast culture media (analogous to extracellular lactate levels) were significantly higher in the *FBXL4^–/–^* human fibroblasts relative to those in controls. By contrast, lactate levels measured in whole worm population tissue homogenates reflect the sum of intracellular and extracellular lactate levels, an important difference that we postulate underlies the observed reduction in the overall lactate level in *fbxl-1*(*ok3741*) worms, where tissue lactate used as a metabolic substrate may potentially be greater in the mitochondrial disease worms relative to controls. Indeed, in humans it is known that lactate serves as an important metabolic intermediate between different tissues and cells (e.g., muscle versus liver, neuron versus astrocyte), where the “lactate shuttle” continually forms and utilizes lactate in different cells under both anaerobic and aerobic conditions (58). Furthermore, generated lactate can be shuttled to tissues such as liver, skeletal muscle, and heart for use as a gluconeogenic precursor or substrate for oxidation (59). While *fbxl4^sa12470^* larvae did not show gross phenotypic abnormalities at baseline, *fbxl4^sa12470^* larvae had significantly decreased complex IV enzyme activity, with liver vacuolization and mitochondrial ultrastructure damage (Figure 5). Under stressed conditions, several major morphological abnormalities developed, including brain atrophy and neurologic and/or muscular impairment, features reminiscent of those seen in patients with *FBXL4-*based mitochondrial disease (2). Slightly bent tails were observed in the CAP-stressed mutant larvae, and further studies will be needed to determine if this observation reflects abnormalities of neurologic tone, muscle function, and/or tissue structural damage. Reduced complex IV enzyme activity in the mutant zebrafish is consistent with the observation that complex IV enzyme activity is one of the most frequently affected RC complexes in patients with *FBXL4*-related diseases (2, 55).

DCA was the only empiric therapy of the 12 drugs screened to significantly ameliorate the reduced brood size of *fbxl-1*(*ok3741*) *C*. *elegans*. Extensive phenotypic characterization further showed DCA improved neurologic and/or muscular function at the level of pharyngeal pump rate and trend toward improved swimming activity in *fbxl-1*(*ok3741*) *C*. *elegans*, prevented brain death and loss of locomotor responses in *fbxl4^sa12470^* zebrafish larvae stressed with CAP, increased the mitochondrial sequelae at the level of mitochondrial function in *C*. *elegans* and *FBXL4*^–/–^ human fibroblasts, and rescued mitochondrial morphology and ultrastructure in *FBXL4*^–/–^ human fibroblasts. By virtue of its ability to activate the PDHc by inhibiting pyruvate dehydrogenase kinase, DCA has long been a candidate therapy proposed, but not widely used, for primary mitochondrial diseases. Activation of PDHc enhances its function as a source for reducing equivalent and Ac-CoA generation in the TCA cycle, which drives oxidative phosphorylation, and can increase the rate of formation of ATP (60, 61). As such, PDHc is a crucial control point for cellular energy homeostasis. Significantly increased brood size after DCA treatment was also previously reported in a *C*. *elegans* complex I mutant (62); this was accompanied by reduced reactive oxygen species production and improved health and life span (63). Studies in both humans and animal models demonstrated that DCA treatment reduced lactate levels under various conditions and may attenuate symptoms in some children with congenital lactic acidosis and affected by various mitochondrial disorders (64–68), although DCA did not affect the lactate levels in the *fbxl-1*(*ok3741*) worms studied here. In addition, DCA was found to stabilize mitochondrial fusion dynamics, survival, and motor performance in models of neurodegenerative diseases (69, 70). In our study, we demonstrated that DCA increases CS activity in both *C*. *elegans* and patient fibroblasts, which we postulate may be an adaptation to PDK inhibition to increase TCA cycle anaplerosis. Furthermore, intracellular lactate was decreased in patient fibroblasts after DCA treatment. The neurologic and/or muscular responses of *C*. *elegans* (increased pharyngeal pump rate and egg-laying ability) and zebrafish (increased touch and tap responses) to DCA treatment is consistent with previous studies that demonstrated the efficacy of short-term treatment of DCA on cerebral metabolism and muscle performance in patients with mitochondrial diseases (71, 72).

However, in a randomized controlled clinical trial, DCA was found to cause toxic neuropathy in humans with mitochondrial encephalomyopathy, lactic acidosis, and stroke-like episodes syndrome (73), although subsequent studies suggested the peripheral neuropathy was related to DCA metabolic rates that could be predicted with dose adjustment on the basis of glutathione transferase ζ-1 genotypes (74). Currently, a multisite phase III clinical trial is underway to study DCA treatment effects in children with primary PDHc deficiency (ClinicalTrials.gov NCT02616484).

While it remains uncertain whether DCA may be a good drug candidate for other primary mitochondrial diseases and will rescue lactic acidemia in all cases, this study demonstrates the apparent tolerability and preliminary efficacy of DCA across 3 evolutionarily distinct models of FBXL4 disease, namely *C*. *elegans*, zebrafish, and human fibroblasts. Collectively, these data suggest that DCA holds promise as a therapeutic lead to improve neurologic and/or muscular function and mitochondrial physiology at the levels of mitochondrial dysfunction and altered morphology. DCA treatment increased CS enzyme activity, which is suggestive of increase in mitochondrial amount, but further investigation will be needed to understand the direct effect of the treatment on mitochondrial content and molecular correlates of mitochondrial morphology. Rigorous clinical trial evaluation is warranted to objectively determine whether DCA will improve the survival, function, and quality of life of humans with *FBXL4* disease.

## Methods

Please see the Supplemental Methods section for further information.

### Study approval.

Approval for study of human participants was obtained per the Children’s Hospital of Philadelphia Institutional Review Board (study 08-6177, MJF, PI). Approval for studies in zebrafish was obtained by the Children’s Hospital of Philadelphia IACUC (21-001154; CS, PI).

## Author contributions

MJF, ML, and ENO conceived of and designed the study. ML designed experimental settings, analyzed data, and performed experiments on *C*. *elegans* and zebrafish. NDM performed the WormScan assay and species protein alignment. EH collected *C*. *elegans* worms for the electron transport chain assay and, together with NS performed the *C*. *elegans* pharyngeal pump assay. NS also performed worm data analysis. ML and CS performed zebrafish studies. ENO devised and performed ATP, lactate, CS, and RC enzyme activity analyses. ML and CS performed and analyzed zebrafish studies. RX advised and assisted with all statistical analyses. SH performed mtDNA analysis in *C*. *elegans*. ML, ENO, and MJF wrote the manuscript. All authors approved of the final version.

## Supplementary Material

Supplemental data

Supplemental video 1

Supplemental video 2

Supplemental video 3

Supplemental video 4

Supplemental video 5

Supplemental video 6

Supplemental video 7

## Version 1 07/26/2022

In-Press Preview

## Version 2 08/22/2022

Electronic publication
