# Supplementary material for: Dichloroacetate improves mitochondrial function, physiology, and morphology in FBXL4 disease models
Source: JCI Insight. 2022 Aug 22;7(16):e156346. doi: 10.1172/jci.insight.156346 (PMC9462489; doi:10.1172/jci.insight.156346)
Supplement: Supplemental data [file jciinsight-7-156346-s236.pdf]

SUPPLEMENTAL MATERIAL

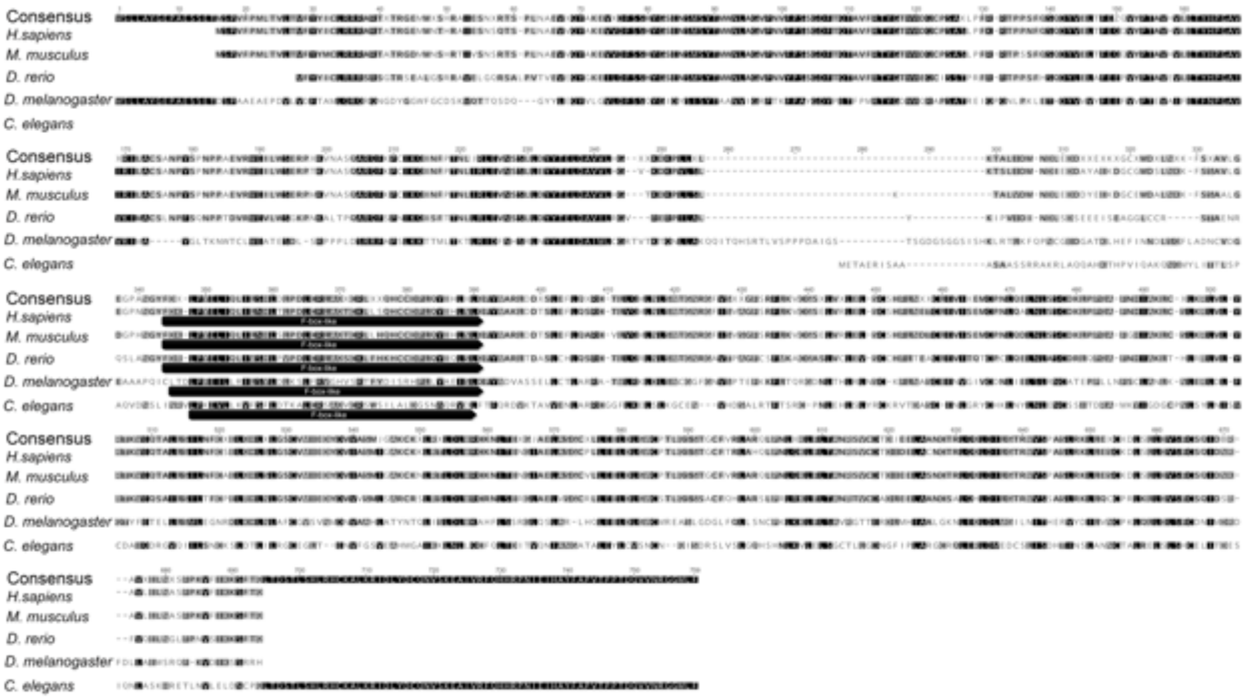

Supplemental Figure 1. Protein alignment of the FBXL4 in *Homo sapiens*, *Mus musculus*, *Drosophila melanogaster*, *Danio rerio* and *Caenorhabditis elegans.* Alignment generated with ClustalW version 2.0.12. Identities are identified as dark type on a white background, nonidentities are white type on a dark background.

Drugs	Concentrations tested by WormScan
Nicotinamide	1, 10 , 100 μM
Riboflavin	100 nM, 1, 10 μM
Glucose	10, 100 μM, 10 mM
Thiamine HCL	1, 10, 100 μM
N- acetylcysteine	1, 10, 100 μM
Folinic Acid	100 nM, 1, 10 μM
Cysteamine HCL	100, 500 nM, 1 uM
AICAR	1, 10, 100 μM
Hydralazine	1, 10, 100 μM
Lithium Chloride	1, 10, 100 μM
Cycloheximide	1, 10, 100 μM
DCA	5, 25, 50 mM

Supplemental Table 1. Table of drugs tested on *fbxl-1(ok3741)* prior dichloroacetate.
Drugs evaluated for their effects on *fbxl-1(ok3741)* brood size and relative concentrations tested are represented.

Supplemental Movie 1. Oblique illumination method to analyze pharyngeal pumping rate.
Example of a one-day adult WT worm observed under a dissecting microscope using the oblique illumination method by sliding the diaphragm to project the light obliquely onto the specimen. The grinder appears as an easily identified bright spot in the head region that is readily followed during contraction.

Supplemental Movie 2. Body bends in WT worms on agar.
During the estimation of body bends, the WT worm constantly moved and only temporarily arrested without completing an entire bend.

Supplemental Movie 3. Body bends in *fbxl-1(ok3741)* worms on agar.
During the estimation of body bends, *fbxl-1(ok3741)* worms showed different behavior on agar compared to WT (see Supplementary Movie 2): the *fbxl-1(ok3741)* worm hesitated while moving and did not complete a maximal body bend.

Supplemental Movie 4. Activity of WT worms in liquid media.

The WT worms' swim activity in liquid media is clearly higher than the activity the mutant worm exhibits (Supplementary Movie 5).

Supplemental Movie 5. Activity of *fbxl-1(ok3741)* worms in liquid media.
The mutant *fbxl-1(ok3741)* worms' activity in liquid media is very low when compared to WT (see Supplementary Movie 4).

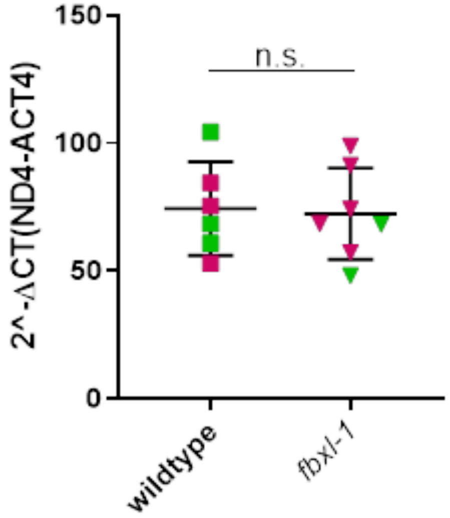

Supplemental Figure 2. mtDNA copy number in *fbxl-1* (*ok3741)* worms. The mtDNA copy number was determined by qPCR using at least six replicates per genotype by adapting a published protocol (Haroon et al., 2018). For each replicate, 10 L4 worms were collected in 5 µL of Extraction Buffer of the Extracta DNA Prep kit from Quantabio (Catalog #95091-002) and incubated at 95°C for 30 minutes. At the end of the incubation, 5ul of the Stabilization Buffer from the kit was added and each sample was diluted with 10 µL of H2O. Subsequently, 5.5 µL of each sample was used to run a TaqMan assay with a Universal Master Mix (cat. no. 444040, Thermo Fisher Scientific), where ND4 (cat. no. 4440043, assay ID: AIFAT8G) and ACT-4 (assay ID: Ce02508047_s1, Thermo Fisher Scientific) assays quantified mtDNA and nDNA content, respectively. mtDNA copy numbers were then normalized to nDNA content. There were six or seven replicates assayed per genotype over two separate experiment and student t-test analysis was used for statistical analysis.

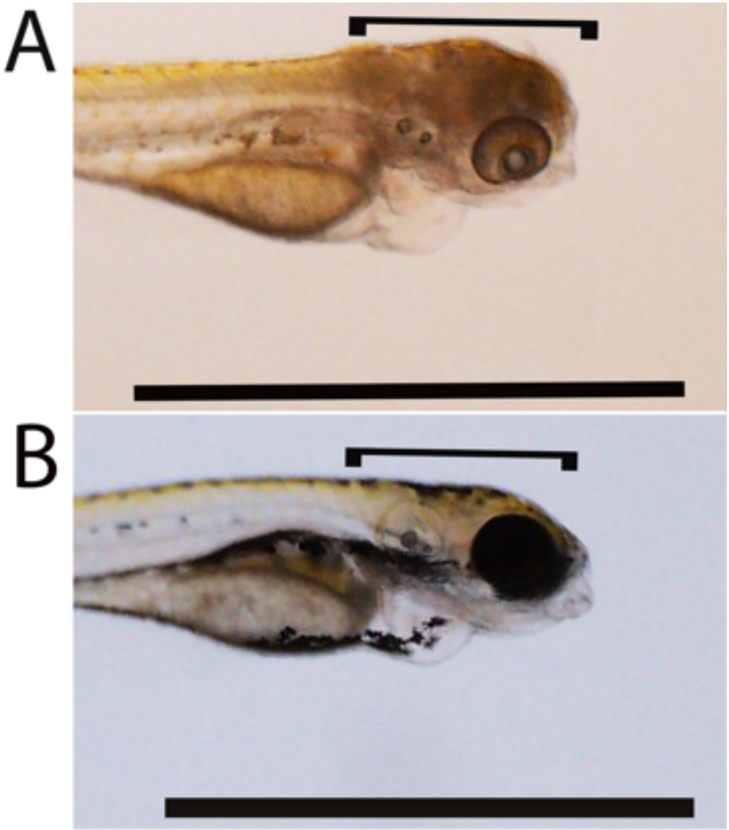

Supplemental Figure 3. High magnification of the gray brain in *fbxl4*^{sa12470} 6dpf larvae. A) Gray brain (bracket) in *fbxl4*^{sa12470} larvae incubated in 2.5 mM CAP for 4 days. **B)** Clear brain (bracket) in *fbxl4*^{sa12470} larvae co-incubated in 2.5 mM CAP and 20 mM DCA for 4 days.

Supplemental Movie 6. Tap response of *fbxl4*^{sa12470} 7dpf larvae incubated in 2.5 mM chloramphenicol. Mutant larvae show slow or absence of neuromuscular tap evoke response.

Supplemental Movie 7. Tap response of *fbxl4*^{sa12470} 7dpf larvae co-incubated in 2.5 mM chloramphenicol and 5 mM DCA. 5 mM DCA treatment improves neuromuscular response in *fbxl4*^{sa12470} 7dpf larvae showing most larvae being able to respond to tap stimuli.

A

% Swim Bladder		
	Stress	Stress + treatment
WT	4.54	6.81
fbxl4^{sa12470}	0	2.22

% Grey Brain		
	Stress	Stress + treatment
WT	2.38	2.72
fbxl4^{sa12470}	71.05	37.77

% Survival		
	Stress	Stress + treatment
WT	97.77	97.77
fbxl4^{sa12470}	84.44	100

% Tap Response		
	Stress	Stress + treatment
WT	95.45	95.45
fbxl4^{sa12470}	26.31	51.11

% Touch Response		
	Stress	Stress + treatment
WT	90.91	90.91
fbxl4^{sa12470}	31.58	84.44

B

WT vs *fbxl4*^{sa12470}					
Conditions	Developmental delay (p value)	Grey brain (p value)	Tap response (p value)	Touch response (p value)	Survival (p value)
Ctrl Media	0.026	1	1	1	1
Stressor	0.5	1.012e-09	2.621e-12	2.084e-09	0.06
WT					
Ctrl Media vs Vehicle	1	1	1	1	1
Ctrl Media vs Stressor	7.287631e-23	1	0.2241379	0.04214559	1
Stress vs treatment	1	1	1	1	1
fbxl4^{sa12470}					
Ctrl Media vs Vehicle	0.7	1	1	1	1
Ctrl Media vs Stressor	2.907765e-20	5.526082e-11	5.378201e-16	1.532787e-14	0.01116396
Stress vs treatment	1	0.034	0.0046	0.00093	0.011

Supplemental Table 2. Percentage and p values of morphological and neuromuscolar statistical analysis of *fbxl4*^{sa12470} and WT larvae after stress and DCA treatment. Stressor indicates 2.5 mM chloramphenicol and treatment indicates incubation of 2.5 mM Chloramphenicol and 5 mM DCA. N = 45 each condition; 3 experiments. **A)** Data (%) obtained from morphological and neuromuscolar analysis of 7dpf *fbxl4*^{sa12470} and WT larvae after incubation with 2.5 mM CAP and and DCA treatment from 2 dpf. **B)** P values obtained from statistical analysis. In red rectangles, significant values are highlighted. Cochran-Mantel-Haenszel chi-squared and Fisher's exact test were used and for comparisons between WT and mutant.

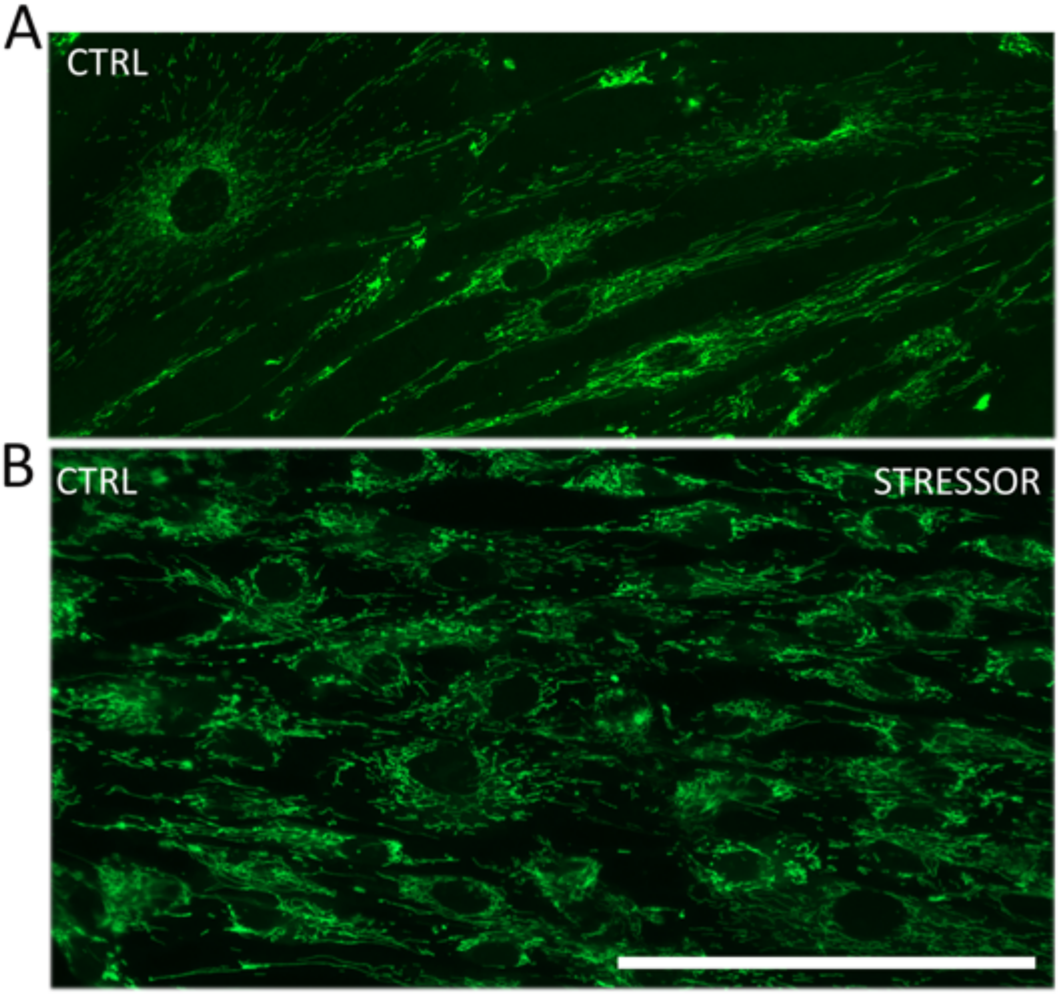

Supplemental Figure 4. Mitochondrial morphology in control fibroblasts. A) Mitochondria (in green) strained with MitoTracker green in control (ctrl) fibroblasts incubated in control DMEM media. B) Mitochondria in CTRL fibroblasts incubated in the stressor media. The stressor did not affect mitochondrial morphology in the control fibroblasts. Scale bar = 200 µm.

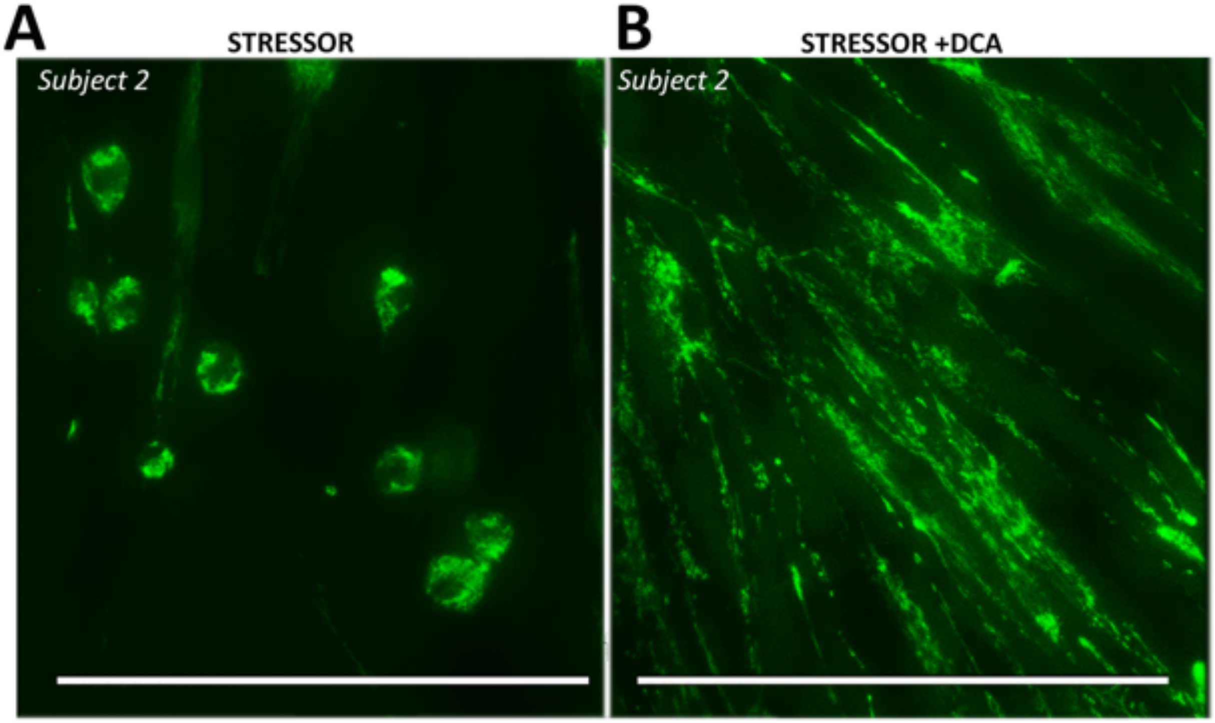

Supplemental Figure 5. DCA efficacy on mitochondrial morphology in subject 2 fibroblasts. A) Mitochondria (in green) strained with MitoTracker green in subject 2 fibroblasts incubated in the stressor (CAP). Mitochondria are fragmented. B) Mitochondria in subject 2 fibroblasts co-incubated in the stressor and 20 mM DCA. DCA treatment improved mitochondrial morphology as demonstrated by more elongated mitochondria in B. Scale bar = 200 µm.

Supplemental Material and Methods

C. elegans **and zebrafish** ***FBXL4***$^{-/-}$ **orthologue strains description and husbandry**. We studied the VC3038 worm strain (1) that harbored a 707 base pair homozygous deletion in *fbxl-1* (*C02F5.7* official gene name*, ok3741* allele*,* g.410_1116del genomic breakpoints, p.Phe105_Lys308del amino acid breakpoints) relative to N2 Bristol worms as wild-type (N2 WT) control. Homozygosity was confirmed by genotyping *C. elegans fbxl-1* using the following primers: 5'-CACCAAAGCCCTCTGTCGAT-3'; 5'-GTTTCACGGTGTTTCGAGGC-3'. *C. elegans* husbandry and all experiments were performed at 20 °C, as previously described (2). *C. elegans* strains were grown on nematode growth medium (NGM) containing OP50 *E. Coli* spread on agar plates.

To reduce the chance of secondary site mutants we outcrossed the worm three times with wild-type N2 as suggested in Zuryn and Jarriault, 2013 (3). *fbxl4*^{sa12470} zebrafish were homozygous for a point mutation c.813T>A that generated a premature stop codon in *fbxl4* (4) and led to the following protein change: p.Y271X, based on NM_001007315. Homozygosity was confirmed using the following primers: 5'-CCACTGCAAACCACACATGT-3'; 5'-GGCCCTCTATAAGATCCCGG-3', and the size of the DNA fragments was quantified after digestion with the AflII restriction enzyme (#R0520, New England Biolab). AB zebrafish were used as controls (AB WT). Zebrafish maintenance and husbandry were performed at 28 °C in the zebrafish aquatic core facility at the Children's Hospital of Philadelphia (CHOP). All protocols and methods were performed in accordance with IACUC #21-001154 regulations for care and use of *D. rerio* at CHOP. Adult zebrafish were set pairwise in undivided mating tanks, embryos were collected and sorted on 0 dpf and placed in embryo water in a 28 °C incubator, as previously described (5).

FBXL4$^{-/-}$ **human fibroblast cell lines genotype and culture.** Primary fibroblast cell lines derived from skin biopsies performed under CHOP IRB #08-6177 (Falk, PI) in human healthy controls and subjects with genetically-confirmed primary RC disease were cultured using standard methods, as previously described (6). Specifically, fibroblasts obtained from two subjects harboring homozygous pathogenic variants in *FBXL4* were studied: subject n.1 (c.[1790A>C; 1067del], p.[Gln597Pro; Gly356fs]) and subject n.2 (c. [618_621dupACTG; c.1641_1642delTG], p. [E208TfsX5; C547X]).

Protein alignment across species. FBXL4 protein alignment was generated with ClustalW version 2.0.12 (7).

Developmental and growth analyses in *fbxl-1(ok3741) C. elegans*. *fbxl-1(ok3741)* and N2 WT and gravid adult worms were selected during each experiment, transferred to an NGM plate with OP50 and allowed to lay eggs for 2.5 h, after which eggs were randomly chosen and transferred to a fresh NGM plate spread with OP50. After 64 h, the developmental stages of both N2 WT and *fbxl-1(ok3741)* worms were analyzed. Biological triplicate experiments were conducted. Worms' stage was synchronized and worms' growth, body length and width were evaluated in each animal upon reaching the 1-day adult stage. Worms were imaged using a stereomicroscope (Nikon SMZ800) and body length and width were measured using Fiji (8) The width was obtained by averaging three measurements analyzed along the worm's body. Five biological replicates were carried out per condition.

Brood size and egg hatching rate analyses in *fbxl-1(ok3741) C. elegans*. Worms' brood size was determined by manually transferring synchronized L4 stage worms (N2 WT, n = 13; *fbxl-1(ok3741),* n = 14) to an NGM plate spread with OP50 *E. coli* (1 worm/plate) and counting progeny over 6 days. Egg hatching rate was estimated in the following way: synchronized gravid 1-day adult worms were selected to lay eggs for 3 h, 136 eggs were randomly chosen and counted, after which hatched larvae were then allowed to grow on NGM plates spread with OP50 until reaching the L3/L4 larval stage when hatching rate was scored by counting the number of worms on the plates. A total of 136 eggs for each strain were selected. Three biological replicates were performed. In addition, brood size was separately evaluated by a semi-automated method, WormScan (9). With this method, both WT and *fbxl-1* brood sizes were estimated in control liquid nematode growth media (LNGM, 50 µL of *E. coli* 2% weight/volume), adjusted from Hirsh *et*

al., 1976 (10). WormScan activity studies were performed at baseline and after treatment of both WT and mutant strains with 25 mM dichloroacetate (DCA, Sigma-Aldrich), a concentration chosen based on results obtained from previous experiments in LNGM that tested 5 mM, 25 mM and 50 mM and showed that 25 mM DCA yielded the greatest efficacy on increasing brood size (data not shown). Worms were treated with 25 mM DCA for 4 days beginning at the synchronized L4 larval stage. L4 stage worms were transferred to a 96-well plate (two worms per well) containing buffer control liquid media or 25 mM DCA in liquid media. Three biological replicates were obtained per condition. Plates were immediately scanned sequentially twice on a flatbed scanner (Epson V800) on day 4, and brood size analysis was obtained by analyzing the pixel difference of skeletonized images. The difference image score was normalized using a "percent of control" method to derive a normalized WormScan score, as previously described (9).

Pharyngeal pumping analysis in *fbxl-1(ok3741) C. elegans*. Pharyngeal pumping rates were measured by manually counting the pharyngeal grinder contractions in the terminal pharyngeal bulb at both 1- and 6-day adult stages**.** Grinder movement was recorded for 3 min using a Leica MS5 microscope and a Cool SNAP cf2 monochrome camera (Photometrics) by obliquely illuminating the worms. N2 WT and *fbxl-1(ok3741)* worms were synchronized and transferred to NGM plates spread with OP50, both with and without treatment with DCA. DCA treatment was tested only in 1-day adult stage worms. DCA was dissolved in S. basal media (5.85 g NaCl, 1 g K_2HPO_4, 6 g KH_2PO_4, and 5 mg cholesterol, in 1 L H_2O) (11) and 400 µL was spread on a 25 mL NGM agar plate with OP50. Worms were incubated with DCA from egg to 1-day young adult stages. Three biological replicate experiments were performed.

Locomotor behavior and activity analyses in *fbxl-1(ok3741) C. elegans*. Several approaches were used to quantify swimming activity and mobility in *fbxl-1(ok3741)* relative to N2 WT worms. Body bends rate analysis on solid media was performed by estimating body bends per minute in synchronized young adults (not gravid) over 3 min. Five biological replicates were conducted, with analysis of a total of 25 worms per strain. Body bends rate analysis in liquid media (thrashing behavior) was analyzed as BBPS over 1 min of activity in S. basal. Worms' activity was recorded using an Olympus MVX10 microscope and an Olympus DP73 camera and analyzed using the wrMTrck plugin for imageJ (12). To avoid any overlap of worms that would compromise the analysis, a modification of the standard technique was made: three 5 µL liquid media drops were pipetted onto a slide, with a single worm transferred into each drop. Four biological replicate experiments were performed, with a total of 36 worms analyzed per strain. A semi-automated approach was used to quantify *C. elegans* swimming behavior in liquid media, where activity of synchronized 1- day adult worms (not gravid) was recorded and analyzed using ZebraLab (Viewpoint) (13). Total activity of 5 worms per liquid drop of S. basal was recorded at each time as one biological replicate experiment (13), for a total number of worms analyzed per biological replicate experiment of 12 N2 WT and 11 *fbxl-1(ok3741)* without treatment and 11 N2 WT and 12 for *fblx-1(ok3741)* worms following DCA treatment. Across all biological replicate experiments, 60 N2 WT and 55 *fblx-1(ok3741)* worms were analyzed without treatment and 55 N2 WT and 60 *fblx-1(ok3741)* worms were analyzed following DCA treatment. Worm activity was recorded using an Olympus MVX10 microscope and an Olympus DP73 camera. Four biological replicates were obtained and data were normalized as percent of N2 WT control.

Morphologic and locomotion analyses in *fbxl4*^{sa12470} zebrafish stressed with chloramphenicol (CAP) and treated with DCA. AB WT and *fbxl4*^{sa12470} zebrafish larvae were exposed to control media (0.1% DMSO, 10 mM Tris in embryo water) (5), 5 mM DCA diluted in control media, 2.5 mM CAP, or 2.5 mM CAP together with 5 mM DCA from 2 dpf until 7 dpf. CAP and DCA concentrations used in this study were chosen after testing 1.5, 2.5 and 5 mM CAP (according to (5)) and 0.5, 5 and 10 mM DCA on zebrafish larvae (data not shown). *fbxl4*^{sa12470} larvae incubated with 2.5 mM CAP manifest a gross disease phenotype without high mortality that would prohibit neuromotor analysis, and incubation with 5 mM DCA showed greater efficacy to rescue the disease phenotype than 0.5 or 10 mM levels. Larval morphology and neuromotor response were analyzed from 5 to 7 dpf with statistical analysis performed on data obtained at 7 dpf. Morphology was analyzed using a stereomicroscope (Olympus MVX10). Muscle performance and function were evaluated in zebrafish larvae using touch and tap-evoked response as locomotion assay involving manual touching of larvae with a probe (touch response) or tapping the culture dish with a probe (tap response) (14) (5). Statistical analysis was undertaken with 3 independent biological replicates per condition, with 15 fish studied per biological replicate to yield a total of 45 larvae for each condition.

***C. elegans*, zebrafish larvae, and human fibroblast cell line sample preparation for biochemical analyses.** Except where noted, worms were analyzed at the 1-day adult stage. Eggs were transferred to NGM plates spread with OP50 (controls) alone or additionally treated with 25 mM DCA. One day adult worms were collected (~1,000 worms per tube) in S. basal solution. After washing three times with S. basal, buffer was removed, worms were flash-frozen in liquid nitrogen, and then stored at -80 °C until later thawed for biochemical analysis. Thirty zebrafish

embryos at 7 dpf were harvested per condition. After washing with E3 buffer twice, zebrafish larvae were flash-frozen in liquid nitrogen, and stored at -80 °C for biochemical analysis. Fibroblasts were maintained in cell growth media (DMEM: 1 g/L glucose, 10% FBS, 50 µg/mL uridine) and treated with 20 mM DCA in cell growth media for 48 h, then trypsinized, washed and collected in PBS. Buffer was removed and cells were immediately flash-frozen in liquid nitrogen and stored at -80 °C until later thawed for analysis.

For ATP and lactate analyses, frozen worm, or cell pellets were homogenized in ice-cold 0.5 M perchloric acid (PCA) by grinding with a motorized pestle, 1 s of sonication, and repeated freeze/thaw cycles in liquid nitrogen with water. After centrifuging at 16,000 × *g* for 15 min, the supernatant was collected and neutralized by adding ice-cold 1 M potassium carbonate. The supernatant was then collected after centrifuging at 16,000 × *g* for an additional 10 min. For respiratory chain (RC) and citrate synthase (CS) spectrophotometric enzyme activity assays, immediately after thawing, worm pellets were treated on ice with proteinase K (1 mg/mL) for 10 min in a mitochondrial isolation buffer (250 mM sucrose, 20 mM Tris-HCl, 3 mM EDTA, pH 7.4) and 5 mM phenylmethylsulfonyl fluoride (PMSF) was then added to inactivate proteinase K. Worms were washed twice and homogenized in the buffer with a motorized pestle, followed by one freeze-thaw cycle. Mitochondrial enriched fractions were obtained by differential centrifugation. Zebrafish larvae were homogenized in 100 µL of a buffer (250 mM sucrose, 20 mM Tris-HCl, 3 mM Na_4EDTA, pH 7.4) by a combination of grinding, 1 s sonication, and then a freeze-thaw cycle in liquid N_2. Mitochondria-enriched larval fractions were obtained by differential centrifugation.

Lactate analysis. Lactate was assayed spectrophotometrically by coupling its oxidation catalyzed by lactate oxidase (LOX) with the concomitant formation of hydrogen peroxide (H_2O_2) which oxidizes (carboxymethylaminocarbonyl)-4,4'-bis(dimethylamino)diphenylamine sodium salt (DA-64) (15), as we previously described (16). Briefly, 5 µL of neutralized PCA extract was added to 155 µL of lactate assay reaction mixture (0.2 mM DA-64, 1 mM EDTA, 0.1% Triton X-100, and 5 U/mL horseradish peroxidase (HRP) in 100 mM HEPES, pH 7.4), mixed thoroughly, and incubated at 37 °C for 3 min. After, 10 µL of LOX (freshly prepared at 2 U/mL) was added to each well and absorbance was monitored at 727 nm every 20 s for 15 min. Lactate concentrations were calculated using standard curves with sodium L-lactate and normalized by protein concentration (estimated by Bradford assay) (17).

ATP analysis. Separation ATP was performed by HPLC at 50 °C using a YMC-Pack ODS-A column (5 µm, 4.6 × 250 mm) preceded by a guard column. Flow rate was set at 0.4 mL/min. The mobile phase was initially 100% mobile phase A (0.1 M sodium phosphate buffer, pH 6.0). Methanol was linearly increased with mobile phase B (0.1 M sodium phosphate buffer, pH 6.0 containing 25% methanol) to 20% phase B over 10 min. The column was washed after each separation by increasing mobile phase B to 100% for 5 min. UV absorbance was monitored at 260 nm with a Shimadzu SPD-M20A. Pertinent peak areas were integrated by the LabSolution software (Shimadzu), quantified using standard curves, and normalized per 1,000 worms.

RC enzymes and CS activities spectrophotometric analyses. RC complex I and complex II enzyme activities were determined by the reduction of 2,6-dichlorobenzenone-indophenol sodium salt (2,6-DCPIP) at 600 nm (ε_{600} = 21 $mM^{-1}cm^{-1}$). The assay buffer for complex I contained 25

mM KH_2PO_4, pH 7.4, 5 mM $MgCl_2$, 3 mg/mL BSA, 25 µM ubiquinone Q1, 5 µM antimycin A and mitochondria-enriched *C. elegans,* zebrafish larvae*,* or fibroblast extract. The reaction was started with 100 µM NADH in the presence and absence of 5 µM rotenone; rates were calculated after subtracting the rotenone-insensitive activities. The assay buffer for complex II activity contained 25 mM KH_2PO_4 pH 7.4, 5 mM $MgCl_2$, 3 mg/ml BSA, 25 µM ubiquinone Q1, 5 µM antimycin A, 5 µM rotenone, and mitochondria-enriched extracts. The reaction was started with 20 mM succinate. complex IV activity was measured by following the oxidation of reduced cytochrome c at 550 nm (ε_{550} = 21 $mM^{-1}cm^{-1}$). The assay buffer contained 25 mM KH_2PO_4 pH 7.4, 5 mM $MgCl_2$, 0.015% n-dodecyl-β-D-maltoside (DDM), 5 µM antimycin A, 5 µM rotenone, and mitochondria-enriched samples. The reaction was started with 15 µM reduced cytochrome c. Complex IV activity rate was calculated as a first order constant. CS activity was measured in an assay medium with final concentrations of 0.1 mM 5,5′-dithiobis-2-nitrobenzoic acid, 0.3 mM acetyl-CoA, 100 mM triethanolamine-HCl buffer (pH 8.0), 0.05% DDM and 0.5 mM oxaloacetate in a 96-well plate, initiated with mitochondria-enriched extracts and the reaction monitored at 412 nm at 30 °C over 15 min. CS activity (nmol/min/mg protein) was calculated as the rate of production of 2-nitro-5-thiobenzoate at 412 nm (ε_{412} = 13.6 $M^{-1}cm^{-1}$)(18) and normalized by protein concentration. Enzyme activities are normalized to protein concentration and then control samples. All spectrophotometric assays were performed at 30 ºC in 170 µL final volume using a Tecan Infinite 200 PRO plate reader.

Mitochondrial morphological and ultrastructural analysis in human fibroblasts. Human fibroblast cell lines were seeded in a 4-chamber 35 mm glass bottom dish, under conditions of metabolic stress involving growth in DMEM, 10% FBS cell media free of glucose and uridine, either with or without 48 h co-incubation with 20 mM DCA. Fibroblasts were then incubated in

200 nM MitoTracker green for 20 min at 37 °C and washed twice with PBS, as previously reported (19). Mitochondrial morphology was analyzed using an EVOS FL AUTO microscope and a 488 nm laser. More than 100 cells were observed across 3 biological replicate experiments in independent chambers. For electron microscopy, human fibroblasts were incubated in the metabolic stress growth media alone or with 20 mM DCA for 48 h in a 60 mm petri dish. Fibroblasts were then washed in PBS, fixed in 6% (vol/vol) glutaraldehyde and 1% formaldehyde in 0.1 M sodium cacodylate buffer (pH 7.4) at room temperature, collected after scraping, and processed for electron microscopy, as previously reported (20-22). Twenty cells were observed per condition, with two biological replicate experiments performed.

Histological and ultrastructural analysis in zebrafish larvae. Homozygous and WT (AB) zebrafish larvae were collected in 4% paraformaldehyde at 6 dpf, embedded in paraffin and processed for histological thin sections at the Pathology Core of the Children's Hospital of Philadelphia and stained with ematoxylin and eosin. Sections were analyzed using the slide scanner Aperio ScanScope CS-O (Leica Biosystems). Homozygous and WT (AB) zebrafish larvae were collected and fixed in 6% (vol/vol) glutaraldehyde and 1% formaldehyde in 0.1 M sodium cacodylate buffer (pH 7.4) at room temperature and processed for transmission electron microscopy as described above and as previously reported (20-22).

Chemicals. All chemicals were purchased from Sigma (St. Louis, MO) unless otherwise stated. All drugs used in this study were dissolved in the appropriate solvent and final concentrations reached through serial dilutions.

Statistical analyses. Statistical analyses were performed using GraphPad Prism 7.04. Data were summarized by mean ± standard deviation (SD), unless otherwise noted, e.g., as mean ± standard error of mean (SEM). Student's *t*-test was used for two-group comparisons of all experiments except for morphology and behavior analyses in zebrafish larvae. Cochran-Mantel-Haenszel test was used for comparisons of different conditions within AB WT or mutant zebrafish larvae, and Fisher's exact test for comparisons between AB WT and mutant zebrafish larvae within the same condition. Bonferroni correction was used to account for multiple testing issue by using a more stringent p-value cutoff, depending on the number of parallel tests.

REFERENCES

1. Thompson O, Edgley M, Strasbourger P, Flibotte S, Ewing B, Adair R, et al. The million mutation project: a new approach to genetics in Caenorhabditis elegans. *Genome Res.* 2013;23(10):1749-62.
2. McCormack S, Polyak E, Ostrovsky J, Dingley SD, Rao M, Kwon YJ, et al. Pharmacologic targeting of sirtuin and PPAR signaling improves longevity and mitochondrial physiology in respiratory chain complex I mutant Caenorhabditis elegans. *Mitochondrion.* 2015;22:45-59.
3. Zuryn S, and Jarriault S. Deep sequencing strategies for mapping and identifying mutations from genetic screens. *Worm.* 2013;2(3):e25081.
4. Busch-Nentwich E KR, Dooley CM, Scahill C, Sealy I, White R, Herd C, Mehroke S, Wali N, Carruthers S, Hall A, Collins J, Gibbons R, Pusztai Z, Clark R, Stemple D.L. . Sanger Institute Zebrafish Mutation Project mutant data submission. *ZFIN Direct Data Submission (http://zfinorg)*. 2013.
5. Byrnes J, Ganetzky R, Lightfoot R, Tzeng M, Nakamaru-Ogiso E, Seiler C, et al. Pharmacologic modeling of primary mitochondrial respiratory chain dysfunction in zebrafish. *Neurochem Int.* 2018;117:23-34.
6. Polyak E, Ostrovsky J, Peng M, Dingley SD, Tsukikawa M, Kwon YJ, et al. N-acetylcysteine and vitamin E rescue animal longevity and cellular oxidative stress in pre-clinical models of mitochondrial complex I disease. *Mol Genet Metab.* 2018;123(4):449-62.
7. Larkin MA, Blackshields G, Brown NP, Chenna R, McGettigan PA, McWilliam H, et al. Clustal W and Clustal X version 2.0. *Bioinformatics.* 2007;23(21):2947-8.
8. Rasband W. ImageJ. *U S National Institutes of Health, Bethesda, Maryland, USA, https://imagejnihgov/ij/.* 1997-2018

9. Mathew MD, Mathew ND, Miller A, Simpson M, Au V, Garland S, et al. Using C. elegans Forward and Reverse Genetics to Identify New Compounds with Anthelmintic Activity. *PLoS Negl Trop Dis.* 2016;10(10):e0005058.
10. Hirsh D, Oppenheim D, and Klass M. Development of the reproductive system of Caenorhabditis elegans. *Dev Biol.* 1976;49(1):200-19.
11. Stiernagle T. Maintenance of C. elegans. *WormBook.* 2006:1-11.
12. Nussbaum-Krammer CI, Neto MF, Brielmann RM, Pedersen JS, and Morimoto RI. Investigating the spreading and toxicity of prion-like proteins using the metazoan model organism C. elegans. *J Vis Exp.* 2015(95):52321.
13. Lavorato M, Mathew ND, Shah N, Nakamaru-Ogiso E, and Falk MJ. Comparative Analysis of Experimental Methods to Quantify Animal Activity in Caenorhabditis elegans Models of Mitochondrial Disease. *J Vis Exp.* 2021(170).
14. Sztal TE, Ruparelia AA, Williams C, and Bryson-Richardson RJ. Using Touch-evoked Response and Locomotion Assays to Assess Muscle Performance and Function in Zebrafish. *J Vis Exp.* 2016(116).
15. Takagi K, Tatsumi Y, Kitaichi K, Iwase M, Shibata E, Nakao M, et al. A sensitive colorimetric assay for polyamines in erythrocytes using oat seedling polyamine oxidase. *Clin Chim Acta.* 2004;340(1-2):219-27.
16. Guha S, Mathew ND, Konkwo C, Ostrovsky J, Kwon YJ, Polyak E, et al. Combinatorial glucose, nicotinic acid, and N-acetylcysteine therapy has synergistic effect in preclinical C. elegans and zebrafish models of mitochondrial complex I disease. *Hum Mol Genet.* 2021.
17. Bradford MM. A rapid and sensitive method for the quantitation of microgram quantities of protein utilizing the principle of protein-dye binding. *Anal Biochem.* 1976;72:248-54.
18. Collier HB. Letter: A note on the molar absorptivity of reduced Ellman's reagent, 3-carboxylato-4-nitrothiophenolate. *Anal Biochem.* 1973;56(1):310-1.
19. Dingley S, Chapman KA, and Falk MJ. Fluorescence-activated cell sorting analysis of mitochondrial content, membrane potential, and matrix oxidant burden in human lymphoblastoid cell lines. *Methods Mol Biol.* 2012;837:231-9.
20. Lavorato M, Franzini-Armstrong C. Practical Solutions to Frequent Problems Encountered in Thin Sections Electron Microscopy. *Microscopy Today.* 2017;25(3):40-5.
21. Lavorato M, Iyer VR, Dewight W, Cupo RR, Debattisti V, Gomez L, et al. Increased mitochondrial nanotunneling activity, induced by calcium imbalance, affects intermitochondrial matrix exchanges. *Proc Natl Acad Sci U S A.* 2017;114(5):E849-E58.
22. Lavorato M, Loro E, Debattisti V, Khurana TS, and Franzini-Armstrong C. Elongated mitochondrial constrictions and fission in muscle fatigue. *J Cell Sci.* 2018;131(23).
